# Re-evaluation of publicly available gene-expression databases using machine-learning yields a maximum prognostic power in breast cancer

**DOI:** 10.1038/s41598-023-41090-9

**Published:** 2023-10-05

**Authors:** Dimitrij Tschodu, Jürgen Lippoldt, Pablo Gottheil, Anne-Sophie Wegscheider, Josef A. Käs, Axel Niendorf

**Affiliations:** 1 Peter Debye Institute for Soft Matter Physics, Leipzig University, 04103 Leipzig, Germany; 2 Institute for Histology, Cytology and Molecular Diagnostics, MVZ Prof. Dr. med. A. Niendorf Pathologie Hamburg-West GmbH, 22767 Hamburg, Germany

**Keywords:** Breast cancer, Tumour biomarkers, Prognostic markers, Prognostic markers, Computational biophysics

## Abstract

Gene expression signatures refer to patterns of gene activities and are used to classify different types of cancer, determine prognosis, and guide treatment decisions. Advancements in high-throughput technology and machine learning have led to improvements to predict a patient’s prognosis for different cancer phenotypes. However, computational methods for analyzing signatures have not been used to evaluate their prognostic power. Contention remains on the utility of gene expression signatures for prognosis. The prevalent approaches include random signatures, expert knowledge, and machine learning to construct an improved signature. We unify these approaches to evaluate their prognostic power. Re-evaluation of publicly available gene-expression data from 8 databases with 9 machine-learning models revealed previously unreported results. Gene-expression signatures are confirmed to be useful in predicting a patient’s prognosis. Convergent evidence from $\approx$ 10,000 signatures implicates a maximum prognostic power. By calculating the concordance index, which measures how well patients with different prognoses can be discriminated, we show that a signature can correctly discriminate patients’ prognoses no more than 80% of the time. Additionally, we show that more than 50% of the potentially available information is still missing at this value. We surmise that an accurate prognosis must incorporate molecular, clinical, histological, and other complementary factors.

## Introduction

Clinicians use a variety of tools to make informed decisions about patient care, including their medical history, pathological characteristics, and molecular biomarkers. Biomarkers are measurable molecular characteristics that allow clinicians to group patients into different categories^1^. Predictive biomarkers predict how a patient will respond to a particular treatment, while prognostic biomarkers indicate a patient’s risk of developing a particular medical condition or experiencing a specific outcome, such as death or metastasis^2^. Gene-expression signatures are biomarkers that are based on genes or groups of genes. Thanks to recent advancements in high-throughput technologies, the analysis and development of gene-expression signatures have gained significant attention. Researchers are using machine learning techniques to develop more powerful gene-expression signatures. However, despite this increased interest and effort, many of the proposed gene expression signatures have failed to perform better than standard clinical and pathological characteristics when it comes to assessing clinical risk^3–6^.

Answering the question of why gene-expression signatures have fallen short of outperforming traditional clinical and pathological characteristics is a critical step in making informed treatment decisions and can have far-reaching implications for cancer treatment and prediction.

The slow progress in developing effective signatures has been attributed to a number of factors, including inconsistent results^7,8^, poor study design^9^, inadequate validation^10^, and the discovery that even a random selection of genes can be prognostic^11^. Additionally, it has been found that some of the most promising signatures lack any meaningful biological connections to the underlying causes of the disease^12^. However, the validity of these factors is not necessarily generalizable across diverse databases or methodological approaches.

With recent progress in machine learning, it has become feasible to generate gene-expression signatures on a large scale using diverse approaches and evaluate their performance across multiple databases. This offers an opportunity to explore whether a shared underlying factor may be impeding the improvement of gene expression signature performance.

This work provides—to the best of our knowledge—the largest evaluation of gene-expression signatures ($\approx$ 10,000 signatures) in breast cancer. Three different approaches for selecting genes are considered to explore a wide range of different gene signatures: random sampling, since it addresses the finding that even random signatures can be prognostic; collecting genes from signatures reported in the literature, because it addresses expert knowledge and functionally related genes; and selecting genes using machine learning, since ensemble algorithms such as random forests can detect nonlinear relations between genes (“Methods” section). These selections are performed on 8 established breast cancer databases, which provide expression values quantified with different methods such as reverse transcription-PCR or DNA microarrays (“Methods” section). Based on these selections, we construct signatures with 9 different machine-learning models. Our results reveal a stunning discovery—a maximum prognostic power of 80% as measured by the concordance index, meaning that these signatures can correctly order patients’ prognoses in no more than 80% of cases. Using a simple simulation, we show that more than 50% of the potentially available information is still missing at this value.

Given the limitations of gene expression signatures, numerous theoretical investigations, simulations, and experimental observations point toward emergent effects, i.e. toward the dominant role of the (host) system over the cell in dictating the behavior of cells in cancer progression^13–17^. Consequently, we posit that the inherent absence of such fundamental information may account for the maximal prognostic power of gene expression signatures.

## Results

**Figure 1:**
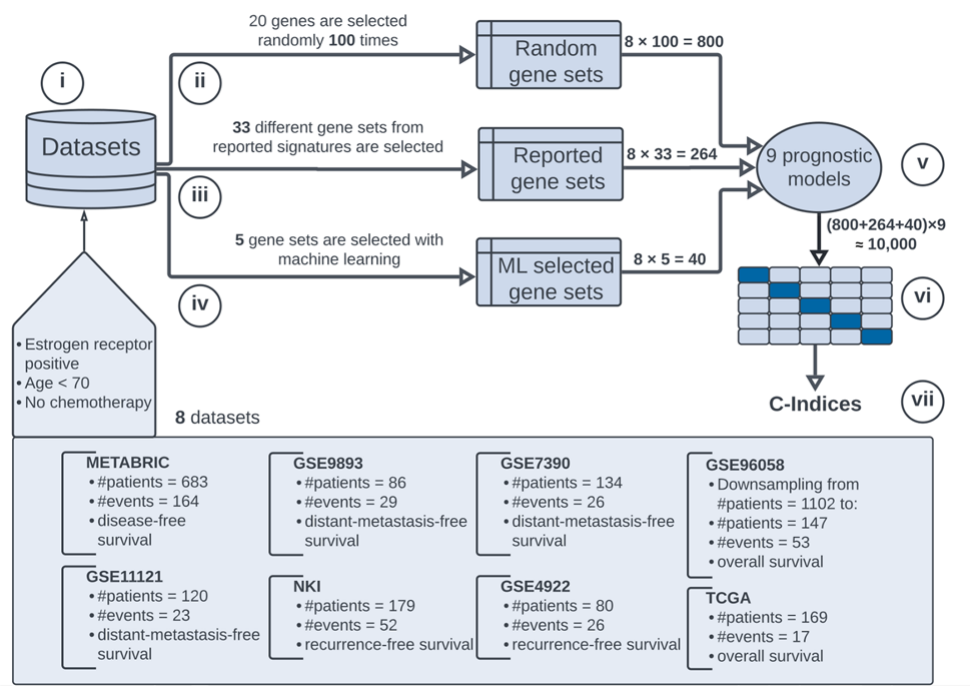
Our 7-step analysis. Steps are indicated in circles. (i) We collect and filter 8 datasets with a different number of patients containing expression data and information about survival (box at the bottom). Filtering is conducted by selecting estrogen-receptor-positive patients under the age of 70 years who did not receive chemotherapy. We use 3 approaches to select gene sets in each dataset: (ii) 20 genes are sampled 100 times at random (Random gene sets), (iii) 33 different gene sets are selected that were reported in the literature (Reported gene sets), (iv) gene sets are selected with 5 machine learning methods (ML selected gene sets). Each gene set serves as input to a prognostic model. (v) Overall 9 prognostic models are developed, resulting in $8\times (100+33+5)\times 9 = 9936 \approx 10{,}000$ signatures. (vi) Evaluation is performed using fivefold cross-validation, whereby each dataset is randomly permuted and split 5 times, each time in 4 training sets and 1 test set (the center-right of the figure, dark blue indicates test set and light blue training set). (vii) Prognostic power is measured by calculating the median C-index based on the 5 cross-validation test sets.

The objective of this study is to evaluate the prognostic efficacy of gene expression signatures and determine their potential maximum prognostic power. The concept of maximum prognostic power is predicated on three fundamental assumptions. Firstly, it is unaffected by the number of patients used for the prognosis, implying that obtaining additional data does not enhance the prognostic ability. Secondly, it is independent of the variable selection technique, meaning that the genes on which the prognosis is based have no bearing on the maximum prognostic power. Finally, it is impervious to the prognostic model or algorithm used to predict survival. To estimate the maximum prognostic power, which endeavors to substantiate these assumptions, we conducted a 7-step analysis outlined in Fig. 1.

(i) Datasets, i.e. expression values and patient information about survival, are collected from 8 different sources. Stemming from different sources, these datasets vary in the number of patients and statistical properties such as primary end-point (“Methods” section), which guarantees that dependency on the number of patients can be examined.
(ii) We select 100 gene sets—a gene set is a list of genes that is used for prognosis—at random in each dataset, resulting in $8\times 100=800$ gene sets. We have determined the optimal number of randomly selected genes in a gene set to be approximately 20 (Supplementary random signature size), which confirms the finding of Chou et. al. (Figure 4 in^18^), who found that 20 genes is optimal by using neural networks. The sampling of random signatures is based on recent studies that emphasize the role of random selections in prognosis^11,19,20^, whereby random signatures outperform published and known signatures. Goh et al.^19^ termed this phenomenon as random signature superiority and related it to the fact that random genes are inseparably correlated with proliferation genes, including genes involved in cell cycle, cell death, contact-based growth inhibition, and so forth.
(iii) Several gene-expression signatures have been reported in the literature and associated with clinical outcome, so that they are expected to provide higher prognostic power than random signatures. We curate 33 gene sets from gene signatures reported in previous studies^21^ such as OncotypeDx, EndoPredict, MammaPrint, or ProSigna, resulting in $8\times 33 = 264$ gene sets. These gene sets are described in detail in SI Appendix, Supplementary reported gene selections.
(iv) Gene sets are chosen with one standard selection method (UM, univariable model) that detects linear relations between genes; and 4 machine learning methods that are based on so-called random forests which can detect non-linearity between genes (“Methods” section), resulting in $8\times 5 = 40$ gene sets.
(v) Prognostic models for each gene set are developed (“Methods” section). A prognostic model provides a statistical function that aims to predict the time from a fixed time point to an event, such as the time from surgery to death, by modeling the relation between one or more variables (genes) and a response (event). The inherent aspect of prognostic modeling is the presence of censored data. For example, a censoring occurs if a patient is lost to followup or the event does not occur within the study duration. We use 9 prognostic models that can handle censored data, resulting in overall $8\times (100+33+5)\times \times 9 \approx 10{,}000$ gene expression signatures. The Cox proportional hazards model^22^ is employed, since it is the most common method for analyzing censored data^23^. However, this model detects only linear effects between variables. In order to detect non-linear effects we use 8 machine learning models based on random forests^24–26^ and so-called gradient boosting machines^27,28^ (“Methods” section).
(vi) Each prognostic model is evaluated using the fivefold-cross-validation, since it can be applied to datasets of different sizes^29^. The validation is typically done using an external dataset or employing a *k*-fold-cross-validation to ensure that models trained on one dataset can be confidently applied to other (external) datasets^29^.
(vii) The median concordance index, also called C-index^30^, is computed. The C-index describes the ability of a prognostic model to separate patients with good and poor outcomes (“Methods” section). C-index of 0.5 denotes a completely random prognosis and a value of 1.0 implies that one can perfectly order the predicted temporal survival probabilities of patients: a patient with a higher survival time would get a higher probability than a patient with a shorter survival time. A C-index = 0 describes the perfect anti-concordance, where the predicted survival probabilities are inversely proportional to the survival times. In his seminal work, Harrell^30^ provides the interpretation of the C-index as the percentage of patients that can be correctly ordered. For instance, a value of 0.7 indicates that one can correctly order patients’ prognoses 70% of all cases.

Steps ii–iv guarantee that the dependency on the selection method can be examined; and step v verifies that the maximum prognostic power is not confounded by a prognostic model. Steps i and v–vii can be considered as an analysis pipeline, where gene selections from different methods (steps ii–iv) serve as input. Thus, we structure our results as follows: In the first three sections we present the computed C-indices for random, reported, and gene sets selected with machine learning. Then the resulting C-indices are brought together according to the three selection methods in order to estimate the maximum prognostic power. Lastly, we provide a measure of how much information is missing at a specific C-index.

However, in order to provide a comprehensive analysis of our results, we conducted a confounding analysis in the Supplementary materials. This analysis focused on investigating the influence of age and clinical variables on prognostic models, serving as a baseline for our main analysis. In our supplementary analysis, we found that when age was used as the sole variable, the average C-index was 0.593 with a standard deviation of 0.063 across all models and datasets used in this study (Supplementary Confounding Analysis, Table 6). Furthermore, we examined the correlation between age and the models’ scores using the METABRIC dataset, our largest dataset, and found that age had no significant correlation with the models’ scores (Supplementary Confounding Analysis, Table 7). Additionally, we evaluated the performance of the gold-standard Nottingham prognostic index^31^, which encompasses relevant clinical variables including the tumor size, grade and the lymph node status, and found that it provided a median C-index of 0.67 across all models used in this study (Supplementary Confounding Analysis, Table 8). These findings further support the robustness and reliability of our analysis.

### The phenomenon of random signature superiority is highly prevalent in breast cancer and can serve as a means of assessing whether gene-expression signatures demonstrate the maximum prognostic power

**Figure 2:**
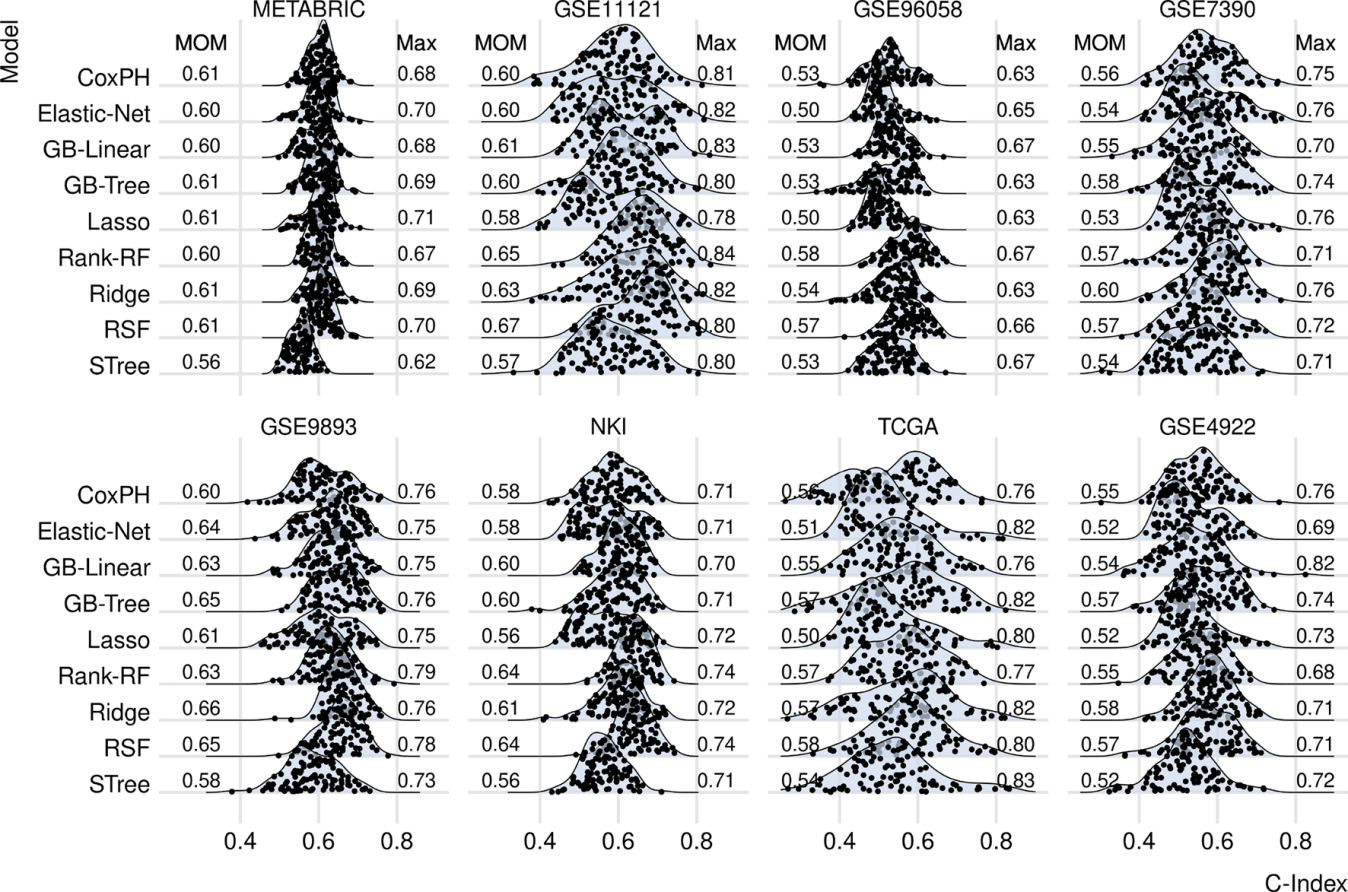
Random signatures: The distributions of C-indices computed using randomly selected gene sets. In total, 100 signatures were evaluated for each prognostic model (row). Each dot represents the median C-index derived from a prognostic model based on a single random selection and calculated via fivefold cross-validation. Each gene set comprises 20 gene expression values. The MOM indicates the median of sample medians, while MAX corresponds to the highest C-index obtained. Each row corresponds to the survival model utilized in the computation, which is described in the “Methods” section, and includes the following: Cox proportional hazards model (CoxPH), Lasso regression (Lasso), Ridge regression (Ridge), elastic net survival regression (Elastic-Net), Gradient boosting with linear learners (GB-Linear) or tree-based learners (GB-Tree), Random survival forests (RSF), maximally selected rank statistics random forests (Rank-RF), and survival trees (STree).

We assessed the prognostic efficacy of gene-expression signatures produced at random, as the concept of random signature superiority (RSS) remains a relatively under-explored field, despite being well-documented^19^. To generate a gene set, we randomly sampled 20 genes from each dataset, resulting in the selection of 100 gene sets. Subsequently, we constructed a prognostic model and computed the corresponding C-index for each gene set. The resulting C-indices are depicted in Fig. 2.

Here, each data point represents the median C-index computed by the fivefold cross-validation and based on a single random gene set. Each row corresponds to a machine learning prognostic model used to compute the risk score. There are 9 prognostic models of 100 random gene sets in each of 8 datasets, resulting in overall $9\times 100\times 8 = 7200$ data points. On the left and on the right sides of each ridge plot, the median of the sample medians (MOM) and the maximum C-index (MAX) are shown, respectively. The median of the sample medians denotes the median value of medians per prognostic model and can be interpreted as the center of the distribution. Additionally, Fig. 2 shows the density plots, which approximate the distributions of the C-indices.

A critical question is whether random signatures are suited to test a potential maximum prognostic power, i.e. whether RSS applies to these data. The signature size is one of the major factors influencing RSS^19^. Thus, to investigate how frequently RSS occurs, we calculated the number of random signatures performing above the C-index of the reported 26-gene signature (which has roughly the same size as random signatures, see Supplementary reported gene selections) for each prognostic model and averaged this value over all datasets. We found (Supplementary random signature superiority) that more than 60% of random signatures outperform the aforementioned reported signature in 4 of 8 datasets, exactly 49% in one dataset, and less than 22% in the remaining 3 datasets. Averaging across datasets, 44% of random signatures outperform the aforementioned reported signature. These results demonstrate that RSS is strongly present in the context of breast cancer gene expression. Consequently, they can be used to test if gene-expression signatures exhibit a maximum prognostic power.

Next, we examined whether the prognostic power can be increased by collecting a larger number of patients. As shown in Fig. 2 the center of the distribution and the variability of C-indices differ across the datasets. For this, we investigated whether the MOM and the median absolute deviation (MAD) correlate with the number of patients as well as with the event rate in a dataset. The event rate is the ratio of the number of events to the number of patients and represents a clinically relevant quantity since prognostic quantities can vary by event rate^32^. These dependencies are plotted in Supplementary dataset dependency for each prognostic model along with the correlation coefficients and their *p* values. As can be inspected there, the MOM and MAD seem to be uncorrelated with both the number of patients and with the event rate. Thus, our data demonstrate that the overall prognostic power cannot be increased by collecting a larger number of patients.

Figure 2 shows also that the best-performing prognostic model is different in each dataset. Consequently, the values of maximum prognostic power are essentially unaffected by the choice of a prognostic model.

The aforementioned points exemplify the utility of random signatures in computing the maximum prognostic power. Despite the highest C-index being 0.84 (GSE11121, Rank-RF) overall, Fig. 2 indicates that the maximum prognostic power appears to hover around a C-index of 0.8 for all models across all datasets.

### As anticipated, the current signatures outperform random signatures: however, they too have limitations and possess a maximum prognostic power

To date, over 30 gene-expression signatures have been reported^21^. Given that these signatures have been linked to clinical outcomes in their respective original studies, it is expected that they will outperform random signatures.

We adopted the approach described in^21^, whose authors searched PubMed for breast cancer signatures or classifiers and collected the gene lists from the original publications. The majority of the corresponding gene sets (28 gene sets) has been used for prognosis, the rest 5 gene sets have been utilized for prediction, i.e. to predict response to a drug. We used these gene lists and the procedure described in Supplementary reported gene selections to select the corresponding gene sets in each dataset.

**Figure 3:**
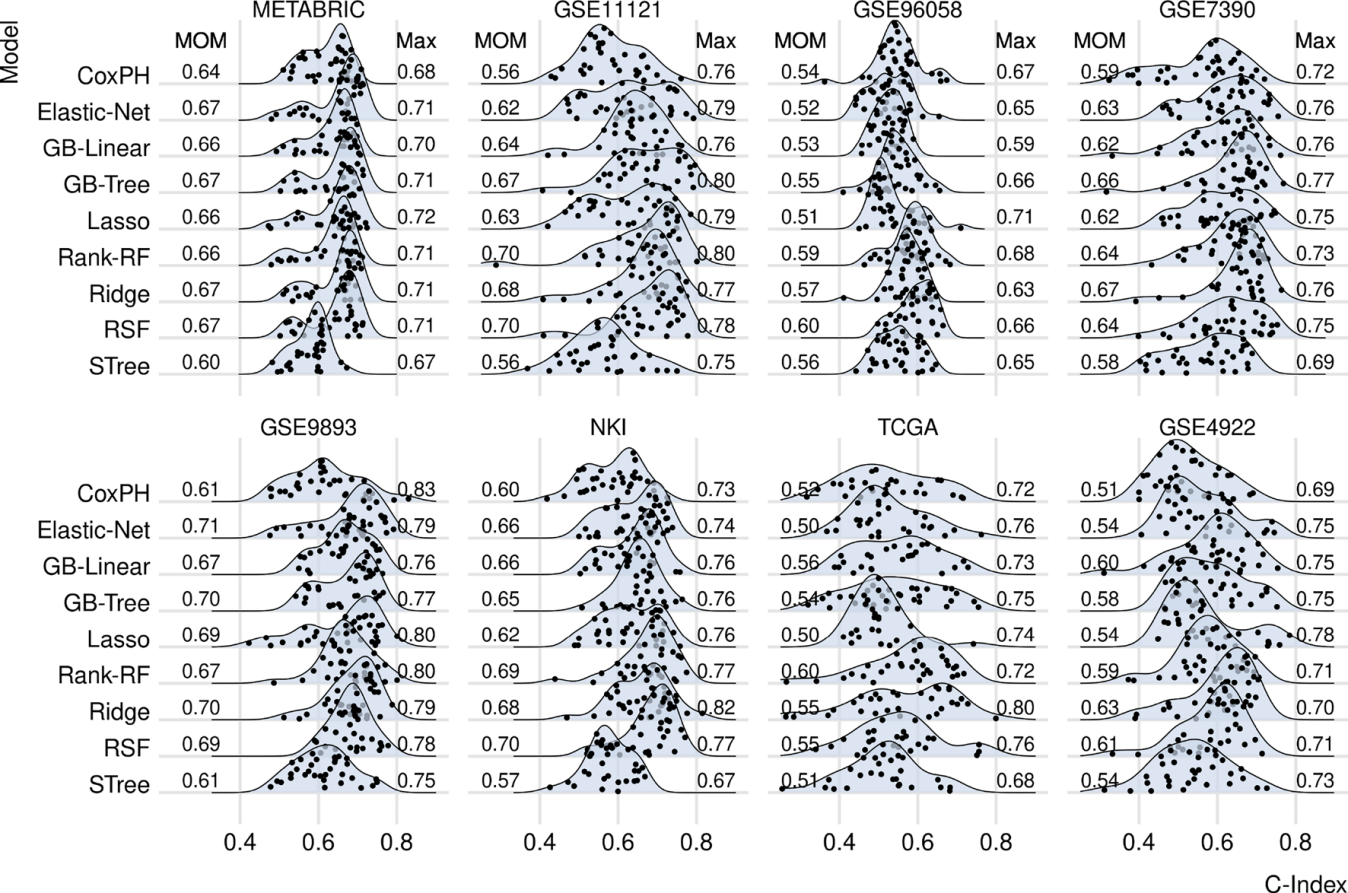
Current signatures: The distributions of C-indices computed based on reported gene sets. Each prognostic model (row) comprises a total of 33 signatures. Each dot represents the C-index derived from a model using a single gene set, which may contain varying numbers of genes. The survival model used for the computation (as described in the “Methods” section) is specified for each row and includes Cox proportional hazards model (CoxPH), Lasso regression (Lasso), Ridge regression (Ridge), elastic net survival regression (Elastic-Net), Gradient boosting with linear learners (GB-Linear), with tree-based learners (GB-Tree), Random survival forests (RSF), maximally selected rank statistics random forests (Rank-RF), and survival trees (STree).

As can be seen in Fig. 3, the C-indices are higher compared with the C-indices of random signatures. To quantify these differences, we show the distributions in form of the violin plots—i.e. box plots showing probability distributions—for each model and each dataset in Supplementary comparison random and reported signatures, and compare the distribution by using the Wilcoxon rank sum test, since the data are not normally distributed. As can be seen, reported signatures tend indeed to have higher C-indices than random signatures, although the level of statistical significance varies across models and datasets.

As already noted for random signatures, the center of the distribution and variability of C-indices differ across the datasets. Similarly, we investigated whether the prognostic power depends on the number of patients and the event rate in a dataset for the reported signatures. As can be seen in the Supplementary dataset dependency, the MOM and MAD are uncorrelated with both the number of patients and with the event rate for reported signatures as well. Thus, these results suggest that the overall prognostic power cannot be increased by collecting a larger number of patients.

Figure 3 corroborates the findings above, indicating that reported gene-expression signatures exhibit an upper limit of C-index around 0.8 across all prognostic models and datasets. Notably, the highest C-index observed is 0.82 (NKI, Ridge).

### Despite the potential of machine learning algorithms to accommodate gene–gene interactions and improve prognostic power, gene-expression signatures developed using these algorithms still demonstrate a maximum prognostic power

Machine learning has the potential to improve prognostic power, since algorithms such as Random Forests have the inherent ability to accommodate interactions between genes^33^. For this reason, we applied 5 state-of-the-art machine learning selection models including Random Survival Forests with variable importance (SRC), with variable hunting (SRC-VH), Minimum Redundancy Maximum Relevance filter (MRMR), and Conditional Variable Importance for Random Forests (CF). The univariable model (UM) serves as baseline model, since it selects only one variable used for prediction of survival (“Methods” section).

**Figure 4:**
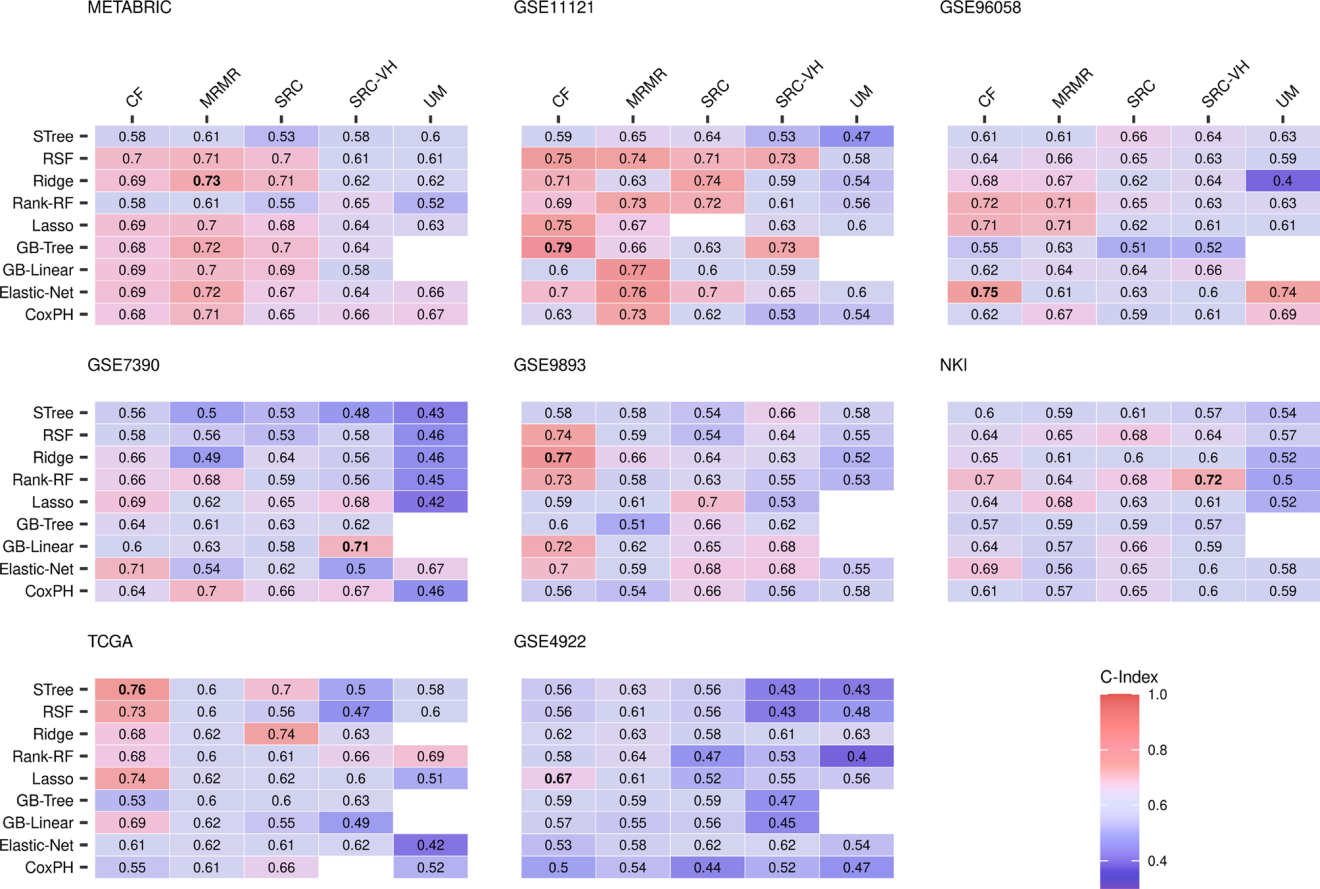
C-indices of gene sets selected with machine learning. Gene sets contain various number of genes (“Methods” section). Each row represents prognostic model used for the computation: Cox proportional hazards model (CoxPH), Lasso regression (Lasso), Ridge regression (Ridge), elastic net survival regression (Elastic-Net), Gradient boosting with linear learners (GB-Linear), with tree-based learners (GB-Tree), Random survival forests (RSF), maximally selected rank statistics random forests (Rank-RF) , and survival trees (STree). Each column represents selection method used for the computation: conditional variable importance for random forests (CF), random survival forests with variable importance (SRC), with variable hunting (SRC-VH), minimum redundancy maximum relevance filter (MRMR), and the univariate model (UM).

The results are given in Fig. 4 in the form of heatmaps that show the C-indices for each combination of machine learning prognostic models (rows) and gene selection methods (columns) for all datasets.

It is evident that no single prognostic model or selection method shows superior performance over the others. These findings suggest that there is no definitive signature that can significantly enhance the prognostic power and serve as a benchmark for other signatures.

We want to clarify that our focus is not on finding the best machine learning model or selection method, but rather to investigate if these results can approach a universal prognostic limit. Nevertheless, it is worth noting that we have explored the impact of the number of patients and the event rate on prognostic power, as we did with random and reported signatures. Supplementary dataset dependency indicates that the MOM and MAD are uncorrelated with both variables, suggesting that collecting more data will not increase the overall prognostic power. Ultimately, the highest C-index across all datasets is 0.79 (GB-Tree, GSE11121), reinforcing the existence of a prognostic limit.

### A comprehensive evaluation yields an inherent prognostic limit for gene-expression signatures

**Figure 5:**
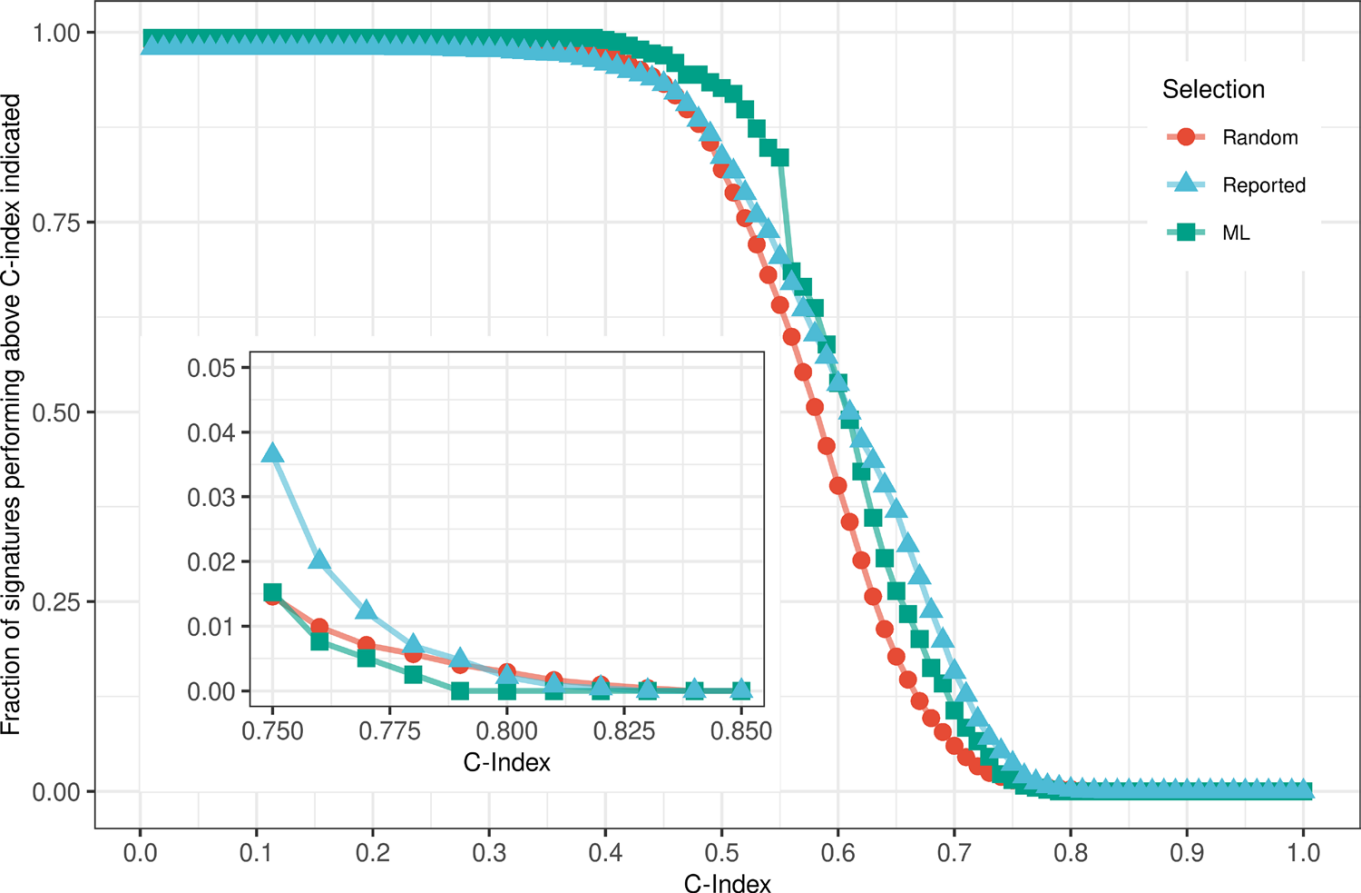
The fraction of gene-expression signatures with a C-index above the threshold indicated on the x-axis, for each selection approach. The gene sets used for the analysis were derived from all 8 datasets and included random, reported, and machine learning-based (ML) selections.

To summarize our findings on prognostic power, we present a graphical representation in Fig. 5 depicting the percentage of signatures across all datasets that exceed the C-index values displayed on the x-axis.

The majority of signatures exhibit a C-index above 0.3 as depicted in the figure. However, the fraction of signatures gradually decreases between this value and 0.4, with a sharp decline observed within the range of 0.4–0.8. The midpoint of this range is approximately at 0.6, with more than 50% of signatures performing above this value.

In the inset of Fig. 5, we focused on the values around 0.8 and observed that the number of gene sets exceeding the C-index of 0.8 is nearly zero. The fraction drops below 1% at the C-index of 0.775 for all gene sets and disappears at 0.825. Hence, we estimate the inherent prognostic limit to be around C-index $\approx$ 0.8. Our comprehensive evaluation, involving 8 datasets, 100 random, 33 reported, and 5 machine learning-based signatures, evaluated by 9 prognostic models, resulted in a total of approximately 10,000 signatures. Based on these findings, it appears unlikely to discover a gene-expression signature that performs better than this limit.

### The current best prognosis method is still missing 50% of the initial information

**Figure 6:**
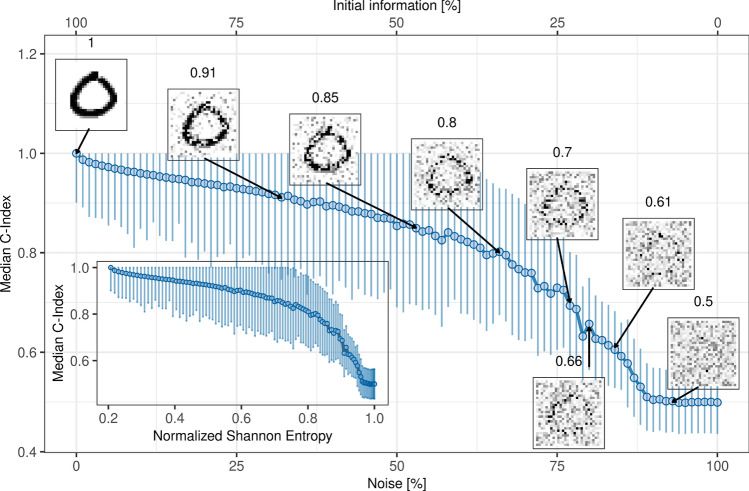
Median C-indices of simulated survival time predictions based on MNIST data. We randomly sample 2000 MNIST images and add varying levels of noise to them. We then reduce the images to 2-dimensional data points, which are used to simulate survival times using Cox proportional hazards models. The C-indices are computed and this process is repeated 100 times, with statistics recorded. The true survival times are based on the simulated survival times of images without noise. The initial information of an image is represented as $100\% - {\text{noise}}\; [\%]$. The number above each exemplary image indicates the calculated C-index of the corresponding image. In the inset, the dependency on the normalized Shannon entropy is shown, with error bars indicating the standard error of the median based on 100 random samples.

To quantify and visualize how much information is missing at a specific C-index, we simulated survival times based on the MNIST data^34^ (“Methods” section), which are 70,000 handwritten $28\times 28$ pixel images of digits ranging from 0 to 9. Hereby, we assigned a survival time to each handwritten digit. We define the initial amount of information of an image as $100\% - {\text{noise}}\; [\%]$. In order to reduce the initial amount of information, we added different amounts of noise ranging from 0 to 100% to the images and computed the C-index at each amount of noise. This process was repeated 100 times, from which the median C-index was calculated. The results are shown in Fig. 6.

As can bee seen, prognosis based on images with 100% noise and with no noise have correctly the C-indices of 0.5 and 1.0, respectively. Common C-indices reported in the literature range from 0.7 to 0.8 (“Methods” section). Interestingly, we see that 75% of the initial information is missing in the middle of this range (C-index = 0.75). From a more practical perspective, one could argue that a C-index of 0.7 is sufficient for prognosis, since one identifies the correct digit from a simple visual inspection. However, more than 60% of initial information is missing at this C-index. Even at the C-index of 0.8 more than 50% of initial information is missing.

An alternative but standard way to look at information gain is the Normalized Shannon entropy^35^, depicted in the inset of Fig. 6: $H(x) = - \sum _{k=1}^{N}p_{k}ln(p_{k})/H_{max}$, where $p_{k}$ is the number of occurrences of the intensity level *k* divided by the number of bins ($28\times 28$ pixels for a MNIST image), *N* denotes the number of intensity levels (which is 256 for a gray-scale MNIST image), and $H_{max}$ is the maximum entropy value.

Thus, *H*(*x*) can be interpreted as the amount of randomness in an image *x*. For example, *H*(*x*) = 0 implies that we know in advance that $p_{k}=1$. Consequently, all pixels will have the same value. A value of 0.2—shown as starting value in the inset of Fig. 6—means that we are 20% uncertain about the information value of the image. On the other hand, a Shannon entropy of 1.0 implies that we 100% uncertain about the information content of an image.

The inset of Fig. 6 shows the dependency of the median C-index on the normalized Shannon entropy. Here, following the direction of decreasing entropy, it is apparent that the C-index increases drastically from 0.5 to roughly 0.8, meaning that in this range the C-index can be largely increased by small amounts of information. In contrast, the C-index rises only steadily above the value of 0.8, hitting a performance plateau, which implies that a prognostic model requires more information gain in order to reach a higher prognostic performance.

## Discussion

Our study uncovers a crucial finding: the existence of a maximum prognostic power inherent to gene-expression signatures. Our comprehensive analysis of over 10,000 gene-expression signatures developed with various methods and tested on multiple established breast cancer databases revealed a maximum prognostic power of up to 0.8 as measured by the concordance index. This means that the ability to correctly order patients’ prognoses is limited to no more than 80% of the time. These results provide a crucial understanding of the prognostic limits of gene-expression signatures, offering a new perspective for future research and clinical practice.

While it is important to consider the limitations of using the C-index as the sole measure of prognostic performance, it is worth noting that this measure is widely accepted and conservative^36^. However, it is closely related to the area under the curve (AUC), which is a commonly used measure in prognostic models. In fact, the C-index can be viewed as an extension of the AUC specifically designed for censored data^36^. In our supplementary analysis (Fig. 10 in *Supplementary Confounding Analysis*), we provide evidence of the association between the C-index and the AUC, along with the Pearson and Spearman correlation coefficients. This analysis demonstrates that our results remain robust and independent of the choice of the prognostic measure.

Our estimate is likely optimistic as we calculated it using fivefold cross-validation, which relied on a similar data distribution due to the resampling of subfolds from the same dataset. However, evaluating predictive performance on external test datasets often leads to a significant drop in performance, a common issue in machine learning research known as dataset shift^37^.

Figures 2 and 3 demonstrate discrepancies in the C-indices among datasets, which may be attributed to inter-platform and inter-cohort variability. However, a more plausible explanation is the use of different event types for prognosis. Disease-free, distant-metastasis-free, and recurrence-free survival predictions are more precise than overall survival predictions, which may encompass events unrelated to the disease. Supporting this explanation is the Supplementary event type, which presents median C-indices in box plots across datasets, showing the lowest performance in datasets utilizing overall survival for prognosis.

It is commonly believed that incorporating more patients in gene-expression signatures would increase their prognostic power. However, our study reveals that the maximum prognostic ability is not influenced by the number of patients. To investigate this, we aggregated 8 datasets with a total of 2500 patients, and resampled the data to obtain different sample sizes ranging from 800 to 2500 patients. We then computed the median of the sample medians of 1000 random signatures for each sample size (Supplementary combined dataset). Our results clearly demonstrate that increasing the number of patients does not lead to improved performance ($p = 0.33$). This conclusion is consistent with the findings of^38^, who observed that adding more data did not enhance prognostic power, albeit using classification error instead of the concordance index. These findings imply that the limitations to better prediction are not due to random noise, but rather stem from a lack of available information.

Our results indicate a notable variance in prognostic ability among algorithms, which may suggest that superior algorithms could improve performance. However, recent research on the use of neural networks with gene expression data has not consistently yielded superior outcomes^39,40^. This suggests that enhancing algorithms alone may not necessarily lead to a better prognosis, further supporting the conclusion that the constraint on performance is probably due to insufficient available information.

It is important to clarify that our study is not intended to identify the optimal prognostic model or to benchmark machine learning models against reported signatures. Significant efforts have already been devoted to exploring interpretable models for patient stratification and biomarker discovery based on molecular knowledge^41–43^. In contrast, the primary objective of our study is to establish an upper limit for signatures, regardless of their origin.

The second major finding of this study is the significant amount of missing information in gene signatures to achieve a prognostic concordance of over 80%. Our analysis of the concordance index in Fig. 6 shows that even at a maximum C-index of 0.8, there is still 50% of missing information. This indicates that we are still far from tapping into all the information needed for an optimal prognosis.

What can be done to overcome the significant lack of prognostic information and improve prognosis?

The study findings suggest that univariable models have the weakest prognostic power, indicating that relying on a single gene expression, clinical, or histological variable is unlikely to capture missing information and accurately predict outcome. To achieve more precise predictions, it may be necessary to refine prognostic models by incorporating multiple complementary factors. Our recent research has demonstrated that a hybrid model that integrates gene expression and clinical information can significantly improve prognostic power^20^. This approach has also been highlighted in various studies on cancer prognosis^44–47^.

Moreover, while past studies have mainly focused on identifying the molecular determinants within the tumor environment^48^, it has become increasingly clear that host factors such as the immune response, dietary variables, or hormone levels can have a significant impact on cell proliferation, invasion, and metastasis. As a result, incorporating these host factors in addition to tumor factors in prognostic models could unlock the missing information and lead to a significant improvement in prognostic power^48^.

The combination of multiple factors can provide more comprehensive information for prognosis, but it remains uncertain if all relevant information can be captured. Machine learning models can detect complex gene relationships, but other factors such as the host’s immune response, diet, and hormone environment also play a role in tumor progression. These interactions may not always be directly related to gene expression, as physical interactions between cancer cells and host tissue can affect cancer cell movement^49^. To address this, a new marker called ”cancer cell unjamming” has been introduced to account for these physical properties in the prognosis process (https://doi.org/10.21203/rs.3.rs-1435523/v1). A holistic approach that considers multiple factors and different scales would be ideal for prognosis to fully understand the emergent effects on higher levels.

In conclusion, our study provides a seminal contribution by offering the first empirical estimation of the maximum prognostic power in cancer. Our findings not only align with previous theoretical works on the limits of predictability in cancer^50–53^ but also address a critical gap in the field. This estimation of maximum prognostic power holds immense value for clinicians, serving as a benchmark to gauge the accuracy of current and future prognostic algorithms. With the deluge of new molecular signature information, need for a comprehensive understanding of the predictability limits is imperative. Our study takes a crucial step in this direction, paving the way for the development of more accurate and dependable prognostic tools for cancer patients.

## Methods

All methods were carried out in accordance with relevant institutional guidelines and all samples used in the METABRIC study were obtained with the consent of patients and appropriate approval from ethical committees (REC ref 07/H0308/161).

### Datasets

The stability of a variable selection method can be affected by changes in the data and varies between datasets, i.e. a selection method can produce gene sets that will be different or invalid when changes to the data occur or a new dataset is used^54^. Thus, gene expressions along with survival data are collected from 8 different datasets.

A dataset can be described as a matrix whose columns contain expression values of thousands of genes and whose rows are organized by tissue samples. The expression values are produced by a quantification method such as reverse transcriptase-PCR, as used for quantification in the Oncotype DX and EndoPredict signatures, or DNA-microarray technology, as used for quantification in the MammaPrint signature. Previous studies have shown that the stability of gene selections varies across different datasets^54,55^. Therefore, we use 8 different but well-established datasets: METABRIC^56^, GSE9893^57^, GSE7390^58^, GSE96058^59^, GSE11121^60^, GSE4922^61^, NKI^62,63^, and data generated by the TCGA Research Network: https://www.cancer.gov/tcga.

The signatures reported in the literature have been proven to work well in hormone receptor-positive breast cancers^21^. Thus, estrogen-positive patients were selected who did not receive chemotherapy to avoid therapy effects as well. Patients above the age of 70 years are disregarded to avoid events due to concomitant diseases.

The number of events and the event used for a specific dataset are shown in Fig. 1. A detailed description of data processing and descriptive statistics of each dataset are provided in Supplementary data.

### Prognostic models

Survival analysis is a statistical treatment of data that aims to predict the time from a fixed time point to an event such as the time from surgery to death. The inherent aspect of survival analysis is the presence of censored data, indicating that the event of interest is never observed in all patients. For example, a patient may be lost to follow-up or the event does not occur within the study duration. The most common method for analyzing these censored data has been the Cox proportional hazards model (CoxPH).

CoxPH model the relationship between the outcome and several variables (also called covariates) by computing the following hazard function:

$$\begin{aligned} h(t) = h_{0} \times \exp \left( \beta _{1}x_{1} + \beta _{2}x_{2} +\cdots + \beta _{n}x_{n}\right) , \end{aligned}$$

which represents the hazard of an event occurring at time point *t* by assigning a (risk) coefficient $\beta _{i}$ to each variable $x_{i}$, whereas $h_{0}(t)$ is the baseline function that is unspecified since it vanishes by dividing the hazards of different patients. By adding the hazards to a time point, the cumulative hazard can be computed and is used to estimate the probability of an event occurring. As can be seen in the equation above, the Cox model assumes that the effects of covariates on survival are additive and constant over time.

However, the Cox model does not generalize well to high dimensional data, where the number of variables exceeds the number of patients. For this reason, machine learning models that extend CoxPH can be applied. The Lasso model (Lasso), Ridge model (Ridge), and the Elastic net model (Elastic-Net) are extensions of CoxPH that incorporate so-called penalties. These penalties are often used to shrink the risk coefficients so that less important variables have less effect in the model. With LASSO penalty a model with a smaller set of coefficients is produced, whereas with Ridge penalty all coefficients are shrunk by the same factor. The Elastic-Net is a linear combination of both penalties and is used to overcome several limitations of Lasso and Ridge (see Supplementary machine learning models).

CoxPH and its penalized extensions impose strong assumptions on the hazard function, that variables are additive and relate multiplicatively to the hazard, as well as that hazard remains constant over time. However, these assumptions are often violated in high-dimensional time-to-event data. To alleviate these problems, algorithms based on random forests and gradient-boosting machines can be used. CoxPH, Lasso, Ridge, and Elastic-Net are single predictive models. Random forests and gradient boosting machines, on the other hand, are ensemble learning algorithms that combine multiple predictive models into an overall ensemble.

For this, original data are resampled by drawing samples with replacement. This procedure is known as bootstrapping. Then a prediction model—called base learner—is applied to each bootstrap sample, and predictions are made by averaging the predictions from the individual base learners. This method is referred to as bagging.

Random forests utilize the bagging of decision trees as base learners. Decision trees are an algorithm that recursively applies a set of yes/no rules to split variables and make predictions based on these splits. However, bagging of decision trees results in tree correlation since samples are drawn with replacement, and thus are partially redundant. Random forests decorrelate decision trees by performing each split on a random subset of the original variables.

Survival trees (STree) and random survival forests (RSF) are extensions of decision trees and random forests to censored time-to-event data, respectively. More specifically, RSF maximizes the survival difference to find the best split of variables. This is done by maximizing the log-rank statistic over all available split points and variables. A major drawback of random forests and RSF is the bias toward selecting variables with many possible split points to splits on, e.g. a variable with a larger variance. Maximally selected rank statistics random survival forests (Rank-RF) overcome this drawback by separating the selection of the variable to split on from the selection of the split point. Instead, a split point is chosen using maximally selected rank statistics, which can also identify non-linear effects in variables.

Whereas random forests combine independent base learners, gradient-boosting machines combine simple base learners, e.g. a decision tree with a few splits, that are trained sequentially in order to improve (boost) the performance of the predecessor. Gradient boosting is trained on the residual errors (gradients) of the entire ensemble at each learning step. Gradient Boosting with linear boosting (GB-Linear) is trained with linear models such as CoxPH as base learners, whereas tree-based boosting (GB-Tree) is trained with decision trees as base learners.

All models and model parameters are described in Supplementary machine learning models.

### Selecting gene sets with machine learning

Variable selection—also called feature selection—is frequently used as a preprocessing to machine learning. It is a process of choosing a subset of original variables in order to remove irrelevant and redundant variables, and thus improve learning performance. In recent years, however, especially gene expression data have become increasingly larger in both the number of patients and a number of variables containing a high degree of irrelevant and redundant information that may greatly degrade the performance of learning algorithms^54^. Therefore, variable selection is necessary for handling high-dimensional data.

We use one standard and four different machine-learning gene selection methods.

The univariable model (UM) uses the univariable CoxPH model that includes just one variable, namely expression values of a single gene, to model the outcome, i.e. the survival of a patient. After each gene in a dataset is modeled to the outcome, gene with the best prognostic performance is chosen.

Random survival forests with variable importance (SRC), with variable hunting (SRC-VH), minimum redundancy maximum relevance filter (MRMR), and conditional variable importance for random forests (CF) use random survival forests to model the outcome but incorporate different measures, i.e. different splitting criteria, for variable importance.

The variable importance of the random forests algorithm (SRC) is computed by permuting the expression values of each gene and calculating the difference between the performances of the prognostic model before and after permutation. Subsequently, the genes are ranked based on these differences and a specified threshold is used to select the most important genes.

The survival random forests variable hunting method (SRC-VH), on the other hand, use a different importance score. First, the standard variable importance, i.e. SRC, is performed. Second, a random subset of genes is selected with probability proportional to calculated variable importance, and a prognostic model is built. Third, the selected genes are ordered by the shortest distance from the tree root to the largest subtree including this gene as its root; they are added successively to the prognostic model until the joint importance does not increase anymore. These steps are iterated a specified number of times. Eventually, the variable importance results from the ranking of the variables based on the frequency of occurrence in these iterations.

The minimum redundancy maximum relevance algorithm (MRMR)^64^ selects variables that are mutually far away from each other, since variables that are mutually close to each other might be redundant. Thus, the algorithm minimizes redundancy by removing the potentially redundant variables. At the same time, the selected variables are highly correlated with the outcome, meaning that they exhibit maximum relevance.

As already mentioned, a major drawback of random forests and RSF is the bias toward selecting variables with many possible outcomes. To address this problem, conditional variable importance for random forests (CF) that utilizes the linear rank statistic as a splitting criterion can be used as well.

All models and parameters used are described in detail in Supplementary gene selection models.

### Concordance index

To assess the prognostic power, we choose the C-index—also called the concordance index or Harrel’s C-index^30,65^—since it is a standard measure for evaluating survival times^36^ and prognostic groups with short-term or long-term survivors can be confidently constructed from the C-index. For instance, it was used by the 2012 DREAM Breast Cancer Challenge that was designed to improve survival prediction (https://doi.org/10.7303/syn1710250^66^). In this challenge, clinical and genomic data of around 2000 patients were available.

The C-index describes the ability of a prognostic model to separate patients with good and poor outcomes. It is a common practice to recall that a C-index of 0.5 denotes a completely random prognosis and a value of 1.0 implies that one can perfectly discriminate predicted patients’ survival probabilities according to their survival times: a patient with a higher survival time would get a higher probability than a patient with a shorter survival time. A C-index = 0 describes the perfect anti-concordance, where the predicted survival probabilities are inversely proportional to survival times.

In his seminal work, Harrell^30^ provides the interpretation of the C-index as a percentage of patients that can be correctly ordered. For instance, a value of 0.7 indicates that one can correctly order patients’ prognoses 70% of the time. Starting from this work, surprisingly, the interpretation of the intermediate C-indices from 0.5 to 1.0 has not been considered more closely^67^. More recently Longato et al.^67^ addressed this problem and proposed a simplified view on the C-index by relating its values to the number of patients whose scores are in the correct order relative to their survival times. Below we provide a further simplified view on the C-index that establishes a relationship between its values and the content of missing information.

### Evaluation of the missing information

To visualize and quantify how much information is missing at a specific C-index, we adapt an idea from^68^ and simulate a scenario, where a 2D image of a handwritten digit corresponds to one patient. We used the MNIST dataset^34^ consisting of 70,000 handwritten $28\times 28$ pixel images of digits ranging from 0 to 9. Then we successively remove information by adding noise to these images, as can be exemplarily seen in the images from left to right above the graph in Fig. 6.

Authors of^68^ simulated survival times based on integer digits ranging from 1 to 4, so that patients with higher digits tend to have shorter survival times. Consequently, their images may represent X-ray images of tumors, with higher digits representing larger, more deadly tumors. In contrast, we choose continuous values in order to model gene expressions.

First, we randomly sample 2000 images, since this number corresponds roughly to the number of patients in a large enough BC gene expression dataset such as METABRIC and the dataset from the DREAM Breast Cancer Challenge.

Second, we reduce these 784-dimensional ($28\times 28 = 784$) images to 2-dimensional continuous data points that can be imagined as expression values of 2 different genes; and simulate survival times. The reduction is done by applying the Principal Component Analysis, which is a technique of reducing high-dimensional data to new uncorrelated variables—called principal components (PCs)—by maximizing variance, i.e. minimizing information loss.

We simulate the survival time *T*(*x*) for each image *x* with the following expression:

$$\begin{aligned} T(x) = {\text{MST}} \cdot \exp ({\text{S}} \cdot \text{(PC1 + PC2)}), \end{aligned}$$

where PC1 and PC2 denote the values of the first and second PCs, respectively. Here, a higher value pf PC1 or PC2 could be imagined as a higher expressed gene.

MST represents the median survival time and S is the coefficient that regulates the skewness of the simulated survival time distribution: the larger this parameter the more right-skewed is the distribution.

To simulate a more realistic survival scenario, we set MST and S to 10 years and to 0.6, respectively, since the value of 0.6 corresponds to most real survival time distributions that are right-skewed.

**Figure 7:**
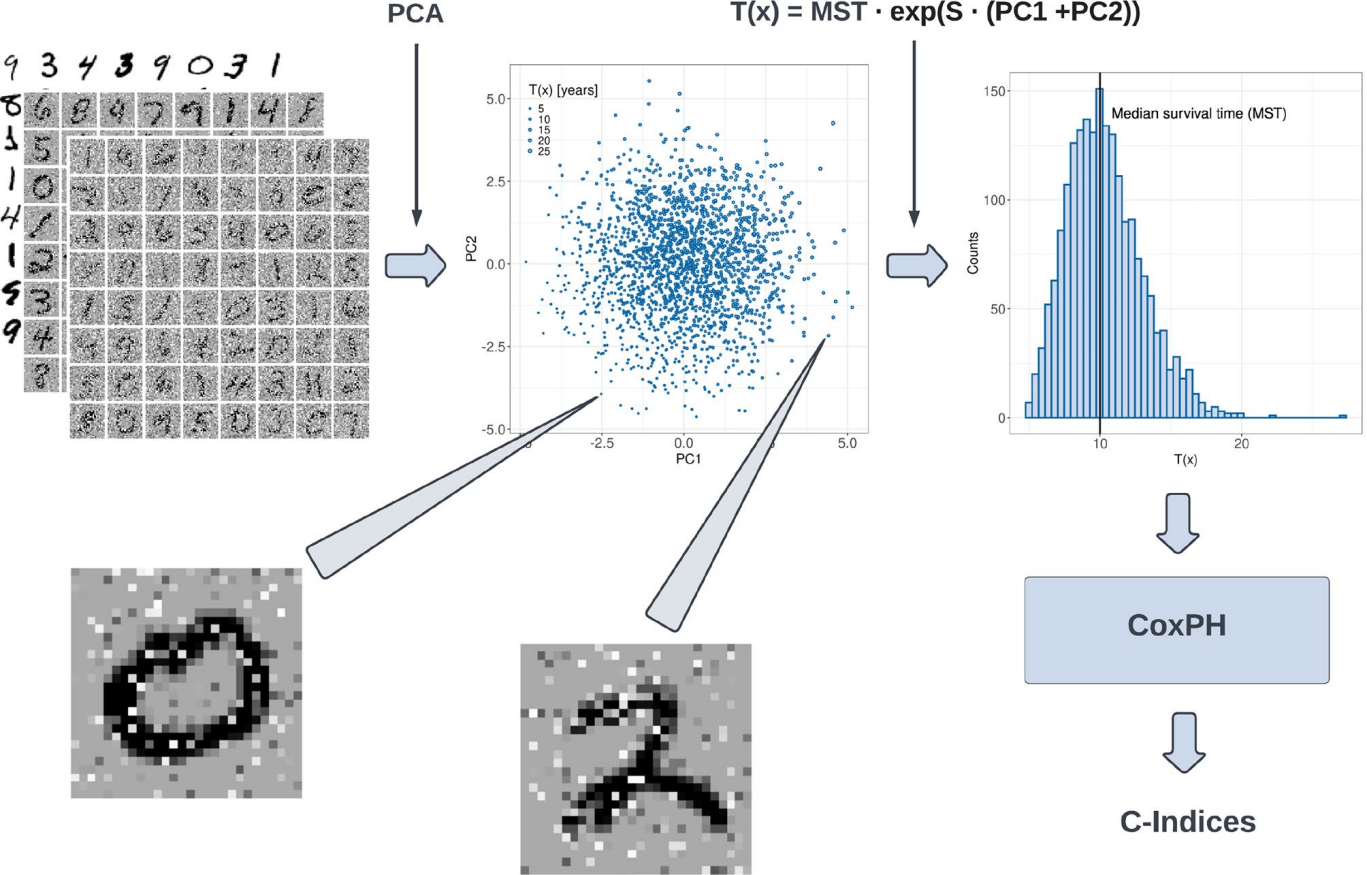
Simulation of survival times. (top left) We sample 2000 MNIST images at random and add different amounts of noise ranging from 0 to 100%. (top middle) For each amount of noise, the sample is reduced to 2 dimensions (PC1 and PC2) using the principle component analysis (PCS). (bottom left and bottom right) Exemplary noised images: each image is reduced to a single point with PC1 and PC2 as coordinates. (top right) Exemplary distribution of survival times based on PC1 and PC2 after applying the equation shown above the distribution. (bottom right). Principal components serve as input variables to the Cox proportional hazards model and simulated survival times as the outcome. Eventually, the c-index is computed at each amount of noise.

In the last step, these reduced data are used as variables in the Cox model, from which the C-index is eventually calculated. This procedure is depicted in Fig. 7.

### Supplementary Information

Supplementary Information.

## Data Availability

All datasets are publicly available. GSE9893, GSE7390, GSE96058, GSE11121, GSE4922, NKI, and TCGA are available in the NCBI Gene Expression Omnibus: https://www.ncbi.nlm.nih.gov/geo/. METABRIC data are available from https://ega-archive.org/dacs/EGAC00001000484 and can be downloaded upon request to EGA (through the METABRIC Institutional Data Access/Ethics Committee; contact via metabric[at]cruk[dot]cam[dot]ac[dot]uk). Code scripts to download, prepare, and analyze data are deposited in a Github repository: https://github.com/DiTscho/LimitOfPrognosis.
