# Supplementary material for: Re-evaluation of publicly available gene-expression databases using machine-learning yields a maximum prognostic power in breast cancer
Source: Sci Rep. 2023 Oct 5;13:16402. doi: 10.1038/s41598-023-41090-9 (PMC10556090; doi:10.1038/s41598-023-41090-9)
Supplement: Supplementary file 1 — Supplementary Information. [file 41598_2023_41090_MOESM1_ESM.pdf]

Supporting information

Dimitrij Tschodu[1,*], Jürgen Lippoldt[1], Pablo Gottheil[1], Anne-Sophie Wegscheider[2], Josef A. Käs[1], and Axel Niendorf[2]

[1]Leipzig University, Peter Debye Institute for Soft Matter Physics, Leipzig, 04103, Germany
[2]MVZ Prof. Dr. med. A. Niendorf Pathologie Hamburg-West GmbH Institute for Histology, Cytology and Molecular Diagnostics, Hamburg, 22767, Germany
[*]dimitrij.tschodu@uni-leipzig.de

Re-evaluation of publicly available gene-expression databases using machine-learning yields a maximum prognostic power in breast cancer

Supplementary Data

For downloading or preprocessing data, Bioconductor packages (https://www.bioconductor.org/) in R (https://www.r-project.org/) were used.

All datasets that are available in the NCBI Gene Expression Omnibus[1] – i.e. all datasets except METABRIC, TCGA, and NKI – were retrieved using the R package GEOquery[2]. If available and if not otherwise mentioned, we downloaded the raw .CEL files, standardized them with the RMA procedure[3], and filtered the data using the WGCNA procedure as described in[4].

We used ComBat in the R package *sva*[5] to adjust data for batch effects.

All scripts to download and prepare data are deposited in a Github repository: https://github.com/DiTscho/LimitOfPrognosis.

In all datasets, estrogen-receptor positive (ER+) patients under the age 70 who did not receive cytotoxic chemotherapy were selected.

In all datasets, genes without annotations were removed.

Datasets

Table 1 summarizes common important clinical parameters across all datasets.

METABRIC

Clinical and pathological annotations as well as gene expression of over 2000 breast cancer tumors were obtained by permission from the METABRIC (Molecular Taxonomy of Breast Cancer International Consortium) consortium[6]. These data can be downloaded from the EuropeanGenome-Phenome Archive at http://www.ebi.ac.uk/ega under accession number EGAS00000000083. The gene expression values were measured on the Illumina HT-12 v3 platform, already preprocessed and log2-normalized, as reported in[6]. The function *avereps* in the R package *limma*[7] was used to summarize genes with multiple probes. The R package *illuminaHumanv3.db* was used to annotate genes[8]. From the initial 2136 samples, we selected 683 samples of patients, who either died due to the disease or are still alive.

TCGA (The Cancer Genome Atlas)

Breast cancer RNA Seq gene expression and clinical data were downloaded from the TCGA website (http://cancergenome.nih.gov) using the package TCGAbiolinks[9]. Gene expressions were filtered and normalized w.r.t. normal solid tissue by the *TMM* method from the *edgeR* R package[10] and the *voom* method[11]. From the initial 1095 samples, we selected 169 using the criteria mentioned above. The overall survival time and status were used for survival analysis.

GSE11121

The datasets contains 200 samples of consecutive lymph node-negative breast cancer patients treated at the Department of Obstetrics and Gynecology of the Johannes Gutenberg University Mainz between 1988 and 1998[12]. From the initial 200 samples, we selected 120 using the criteria mentioned above. The distant-metastasis-free survival time and status were used for survival analysis.

Table 1. Descriptive statistics of common important clinical parameters for all datasets used in this study.

	GSE11121 (N=120)	GSE7390 (N=134)	GSE96058 (N=147)	METABRIC (N=683)
age				
- Median	58.000	47.000	64.000	58.140
- Q1,Q3	48.000, 63.250	42.250, 51.000	56.500, 67.000	50.255, 63.845
grade				
- 1	18 (15.0%)	29 (22.0%)	39 (26.5%)	89 (13.5%)
- 2	86 (71.7%)	68 (51.5%)	81 (55.1%)	329 (49.9%)
- 3	16 (13.3%)	35 (26.5%)	27 (18.4%)	241 (36.6%)
tumor size				
- Median	1.800	2.000	1.600	2.000
- Q1,Q3	1.400, 2.325	1.600, 2.500	1.200, 2.200	1.560, 2.700
node status				
- 0	120 (100.0%)	134 (100.0%)	101 (68.7%)	433 (65.0%)
- 1	0 (0.0%)	0 (0.0%)	40 (27.2%)	233 (35.0%)
- NA	0 (0.0%)	0 (0.0%)	6 (4.1%)	0 (0.0%)
survival time in years				
- Median	7.583	12.731	4.071	11.633
- Q1,Q3	5.500, 10.729	7.764, 15.504	3.110, 5.263	7.001, 17.683
event				
- 0	97 (80.8%)	108 (80.6%)	94 (63.9%)	519 (76.0%)
- 1	23 (19.2%)	26 (19.4%)	53 (36.1%)	164 (24.0%)
	GSE4922 (N=80)	GSE9893 (N=86)	NKI (N=179)	TCGA (N=169)
age				
- Median	57.000	60.550	46.000	61.038
- Q1,Q3	48.750, 66.250	55.325, 65.650	41.000, 50.000	51.712, 64.882
grade				
- 1	33 (41.2%)	15 (18.1%)	52 (29.1%)	0
- 2	39 (48.8%)	55 (66.3%)	67 (37.4%)	0
- 3	8 (10.0%)	13 (15.7%)	60 (33.5%)	0
tumor size				
- Median	1.700	2.000	2.000	NA
- Q1,Q3	1.200, 2.200	1.600, 2.500	1.500, 2.500	NA
node status				
- 0	79 (98.8%)	46 (54.8%)	131 (73.2%)	109 (64.9%)
- 1	1 (1.2%)	38 (45.2%)	48 (26.8%)	59 (35.1%)
survival time in years				
- Median	10.292	5.496	6.521	0.197
- Q1,Q3	5.688, 10.771	4.681, 6.567	4.711, 9.749	0.132, 0.366
event				
- 0	54 (67.5%)	57 (66.3%)	123 (70.3%)	152 (89.9%)
- 1	26 (32.5%)	29 (33.7%)	52 (29.7%)	17 (10.1%)

GSE96058

The available expression matrix contained preprocessed log2-normalized expression values of a prospective population-based series of 3,273 BC patients with a median follow-up of 52 months (Sweden Cancerome Analysis Net- work—Breast [SCAN-B], ClinicalTrials.gov identifier: NCT02306096), as described in[13]. No further standardization was conducted. From the initial 3,273 samples, we selected 1102 using the criteria mentioned above. We subsequently performed downsampling due to a low amount of events ($< 5\%$). For this, a subset of patients was randomly sampled with an event-to-patients-at-risk ratio of roughly 1:3. The overall survival time and status were used for survival analysis.

Data: A .csv file containing already standardized expression data.

GSE4922

In this dataset, two separate breast cancer cohorts can be accessed: the Uppsala (n=249) and the Singapore (n=40) data[14]. Thus, from the initial 289 samples, we selected 80 using the criteria mentioned above. The recurrence-free survival time and status were used for survival analysis.

GSE7390

Gene expression data of frozen samples from 198 lymph-node negative systemically untreated patients were collected at the Bordet Institute[15]. From these data we selected 134 using the criteria mentioned above. The distant-metastasis-free survival time and status were used for survival analysis.

GSE9893

This dataset contains samples from a cohort of 132 primary tumors from tamoxifen-treated patients whose expression profiles were conducted at the whole genome level by 70-mer oligonucleotide microarrays containing 22,680 probes[16]. From the initial 132 samples, we selected 86 using the criteria mentioned above. The distant-metastasis-free survival time and status were used for survival analysis.

Data: Already standardized ExpressionSet.

NKI

These are gene expression data of breast cancer tumors collected at the Netherlands Cancer Institute, as described in[17] and[18]. The R package *BreastCancerNKI* (`https://bioconductor.org/packages/breastCancerNKI/`) was employed to download the data. From the initial 337 samples, we selected 179 using the criteria mentioned above. The recurrence-free survival time and status were used for survival analysis.

Data: Already transformed ExpressionSet from *BreastCancerNKI*.

Supplementary Reported Gene Selections

The authors of[19] collected gene lists of 33 reported signatures in breast cancer. They inquired PubMed for breast cancer gene signatures or classifiers and collected the lists of gene names from the original publications. As a result, they prepared gene lists containing the HUGO gene symbols. These lists and a detailed description of their procedure can be found in Additional Files in their publication[19].

We downloaded these lists and adopted their procedure: not only several studies used different gene alias names, but the used gene names differed also across datasets. Thus, we identified all gene names, i.e. aliases, as official gene symbols individually in each dataset using the R package *org.Hs.eg.db*[20].

For all datasets, since some gene names were missed in a particular dataset, we also identified the most coexpressed genes in this dataset by querying COXPRESdb: a database of coexpressed genes[21]. Table 2 shows the resulting number of extracted genes for all datasets. In the first column, the full names of reported signatures are listed. We adopted the exact gene names of reported signatures from Additional file 2 in[19]. The second column provides the number of genes used in the signatures. Other columns provide the number of extracted genes for each dataset. As mentioned in[19], within some signatures the number of reported genes is less than the number of extracted genes, since these genes are duplicated with different probe names. For the sake of completeness, we decided to include all gene lists into our analysis.

All signatures except the following five signatures are used for prognosis: The GCN of MET and HGF, 28-gene expression profile, 92 predictor, 85-gene signature, and 512-gene signatures are used for prediction, i.e. to predict the response to treatment or drug.

Supplementary Machine Learning Models

Various machine learning models have been adapted or developed to handle censored data. These models can be divided into feature selection models as well as prognostic models. Some prognostic models already include one or more selection models

Table 2. Gene lists from published signatures considered in the analysis.

Signature	gene #	METABRIC	GSE11121	GSE96058	GSE7390	GSE9893	NKI	TCGA	GSE4922
B-cell:IL8 ratio[22]	22	7	11	9	11	9	5	14	10
Breast cancer index[23]	7	7	7	7	7	7	7	7	7
Cell cycle pathway signature[24]	26	26	26	26	26	26	26	26	25
92-gene predictor[25]	92	80	80	80	80	80	80	80	78
EndoPredict assay[26]	8	8	8	8	8	8	8	8	8
GCNs of MET and HGF[27]	2	2	2	2	2	2	2	2	2
8-gene genomic grade index[28]	4	4	4	4	4	4	4	4	4
97-gene genomic grade index[29]	97	87	87	87	87	68	86	87	83
158-gene HER2-derived prognostic predictor[30]	158	152	151	152	151	152	152	152	151
HOXB13:IL17 BR ratio[31]	2	2	2	2	2	2	2	2	2
186-invasivenessgene signature[32]	186	151	150	153	150	149	150	152	145
IHC4 Score[33]	4	4	4	4	4	4	4	4	4
7-gene immune response module[34]	7	6	6	6	6	6	6	7	6
85-gene signature[35]	85	50	50	50	50	50	50	50	49
54-gene lung metastasis signature[36]	54	54	54	54	54	54	54	54	52
MAGE-A[37]	2	2	2	2	2	1	1	2	2
70-gene signature[17]	70	61	61	61	61	60	61	62	61
368-gene medullary breast cancer like signature[38]	368	359	354	363	354	336	350	361	336
14-gene metastasis score[39]	14	14	14	14	14	14	14	14	13
Multigene HRneg/Tneg signature[40]	14	14	14	14	14	14	14	14	14
26-gene signature[41]	26	19	18	19	18	17	18	19	18
264-gene signature[41]	264	210	206	210	206	186	198	211	203
512-gene signature[42]	512	352	350	353	350	345	349	353	343
32-gene p53 status signature[43]	32	19	19	19	19	19	19	19	19
PAM50 assay[44]	50	50	50	50	50	49	50	50	49
64-gene expression signature[45]	64	48	48	48	48	46	48	48	47
127-gene classifier[46]	127	123	123	124	123	112	123	124	118
21-gene signature[47]	16	16	16	16	16	16	16	16	16
26-gene stroma-derived prognostic predictor[48]	26	25	25	25	25	24	25	26	24
8-gene score[49]	8	8	8	8	8	8	8	8	8
T-cell metagene[50]	50	46	46	48	46	46	47	46	44
28-gene expression profile[51]	28	24	24	24	24	23	24	24	24
76-gene signature[52]	76	67	67	68	67	66	66	69	67

in the process of training. Below we provide a short description of the machine learning models used in our study. The Cox proportional hazards model – while not a machine learning model – is used as a baseline.

The Machine Learning in R package (*mlr*)[53] was employed to benchmark models and perform cross-validation. All results are based on 5 repeats of 5-fold cross-validation. All results below correspond to the tuned number of features: We tuned the number of features during each cross-validation fold so that a tuned (optimal) number of features was used for the eventual prediction. Other hyper-parameters are listed in Table 3.

Cox proportional hazards model

The Cox proportional hazards model can be regarded as the standard model for analyzing survival data[54]. In this model, the effect of variables – also called covariates $x_1, x_2, ..., x_n$ – on the time to an event of interest is evaluated. For example, an event might be the death of the patient or a relapse of the disease. Formally, the Cox model is expressed by the following hazard function:

$$h(t) = h_0 \times \exp(x_1\beta_1 + x_2\beta_2 + ... + x_n\beta_n),$$

where $\beta_1, \beta_2, ..., \beta_n$ of n patients denote the regression coefficients, i.e. weights of the covariates: the larger the coefficient the larger effect its covariate has on the prognosis of survival times. They are estimated by maximizing the partial likelihood. The baseline hazard function $h(t)$ remains unspecified since it is divided out by computing the proportional hazard.

The Cox model remains a highly robust model if applied to linearly independent data and under the assumption that the proportional hazard does not change over time. However, this model loses its robustness when applied to high-dimensional data.

Lasso, Ridge, and Elastic-Net Regressions

Since the Cox model generalizes poorly to high dimensional data, some penalizing constraints are often used in the process of maximizing the partial likelihood. As a consequence, the regression coefficients shrink toward zero, their variances reduce as well, and the less important covariates tend to have less effect in the model.

L1 and L2 regularizations are two standard forms of regularization:

$$L1 = \lambda \times (|\beta_1| + |\beta_2| + ... + |\beta_n|),$$
$$L2 = \lambda \times (\beta_1^2 + \beta_2^2 + ... + \beta_n^2),$$

where λ is the regularization constant. The L1 regularization is also known as LASSO regression and produces models with a smaller set of coefficients since several coefficients are completely reduced to zero. Thus, variable selection is also performed during the fitting process.

The L2 regularization is also known as ridge regression and shrinks all coefficients by the same factor. As a result, all coefficients are reduced but none is eliminated.

The L1 regularization cannot select more variables than the number of samples. Moreover, it is biased toward the selection of groups of correlated variables[55].

To overcome these limitations, one can use a linear combination of L1 and L2 penalties, which is then called the **elastic net regression**. The elastic net regression is especially useful when the number of variables is larger than the number of samples[55]. In our study, we evaluated the extensions of the lasso, ridge, and elastic net regressions to the Cox model[56,57].

Boosted models

Boosting is an ensemble learning technique that combines the so-called weak i.e. base learners into stronger learners that are trained sequentially[58]. During each iteration, a new model is added to the ensemble correcting the errors of the previous model. Boosting has been adapted to survival analysis[59,60].

We used the gradient boosting in our study[61]. This type of boosting trains on the residual errors (gradients) of the entire ensemble model at each step. It can also be trained with linear models as base learners as well as with decision trees as base learners. In this study, both methods were assessed.

Survival Trees and Random Survival Forests

Survival trees[62] and random survival forests[63] are an extension of decision trees[64,65] and the random forests algorithm developed by Leo Breiman[66] to censored survival data. Decision trees and random forests, in turn, are nonparametric regression and classification methods that are well suited for the case, where the number of variables is greater than the number of samples, for example in genetics.

Survival Trees

In the general description of decision trees, the space spanned by predictor variables, i.e. by the covariates, is recursively partitioned into several groups such that observations with similar responses are grouped together. In the case of numeric variables such as gene expression values, binary splits are conducted. For the splitting a variable and selecting a splitting threshold, decision trees follow the principle of impurity reduction. Following this principle, each split in the tree results in daughter nodes whose impurity is reduced in comparison to the parent nodes. The impurity can be measured with the Shannon entropy or the Gini index or other statistics. Finally, in an ensemble of trees predictions are made by means of averaging and combining the results of each decision tree.

Random Forests

Random survival forests aggregate the results from ensembles of decision trees, whereas each tree is generated from a bootstrap sample of the data. At each node, a random subset of predictor variables is sampled and one variable is selected to split on. The selected variable maximizes the difference in survival between daughter nodes. Mathematically, the log-rank statistic over all available split points and variables is maximized. For prediction, an average over the predictions of the single trees is used (a vote is used for a classification problem).

Both survival trees and random survival are able to robustly handle high-dimensional non-linear data and detect interactions among them. Provided the depth of trees is chosen carefully, they also reduce the tendency of overfitting the data. However, both algorithms are biased towards selecting more heterogeneous variables, i.e. variables with many possible split points are preferred. To overcome this problem, one can use the conditional inference forests[67] that select the split points based on linear rank statistics. Nonetheless, to detect non-linear effects in the predictor variable space, selecting the split points using maximally selected rank statistics can be conducted[68].

Alongside survival trees, we evaluated both the standard random survival forests and the maximally selected rank statistics random survival forests.

The full names of the prognostic models, respective hyper-parameters as well as packages and functions used in this study are shown in Table 3.

Supplementary Gene Selection Methods

During variable selection, a subset of for the survival outcome relevant variables is selected. We applied 5 different gene selection methods and measured the performance of the survival models described above.

In a univariate model, a univariable Cox proportional hazards model is fitted to the expression values of each gene and the genes are ranked by the resulting C-index of the corresponding model.

Table 3. Machine learning prognostic models and respective hyper-parameters used in this study.

Survival model	Full name of the survival model	Package and function	Hyper-parameters
CoxPH	Cox proportional hazards model	survival, coxph	
Lasso	Lasso regression	glmnet, cv.glmnet	alpha = 1, nfolds = 5
Ridge	Ridge regression	glmnet, cv.glmnet	alpha = 0, nfolds = 5
Elastic-Net	Elastic net survival regression	glmnet, cv.glmnet	alpha = 0.5, nfolds = 5
GB-Linear	Gradient boosting with linear learners	mboost, gamboost	baselearner = "bols"
GB-Tree	Gradientboosting with tree-based learners	mboost, gamboost	baselearner = "btree"
RSF	Random survival forests	randomForestSRC, rfsrc	mtry: from (number of genes)/3 to 100<br>nodesize: 5 to 30, ntree=500
Rank-RF	Maximally selected rank statistics random forests	ranger, ranger	splitrule = "maxstat",<br>importance = "permutation"
STree	Survival trees	rpart, rpart	

The variable importance of the random forests algorithm is computed by permuting the column containing the expression values of each gene and calculating the difference between the performances of the survival model before and after permutation. Subsequently, the genes are ranked based on these differences.

The survival random forests variable hunting method, on the other hand, use a different importance score. First, the standard variable importance is conducted on the entire dataset. Second, a random subset of genes is selected with probability proportional to the calculated variable importance, and a forest is fitted. Third, the selected genes are ordered by the shortest distance from the tree root to the largest subtree including this gene as its root; they are added successively to the fitting model until the joint importance does not increase anymore. These steps are iterated a specified number of times. Eventually, the variable importances result from the ranking of the variables based on the frequency of occurrence in these iterations.

The Minimum Redundancy Maximum Relevance algorithm[69] selects variables that are mutually far away from each other: variables that are mutually close to each other might be redundant. Thus, the algorithm minimizes redundancy by removing the potentially redundant variables. At the same time, the selected variables are highly correlated with the response variable such as survival time, meaning that they exhibit maximum relevance.

The Conditional Variable Importance for Random Forests utilizes the linear rank statistics of conditional random forests described above.

The full names of the selection methods, respective hyper-parameters as well as packages and functions used in this study are shown in Table 4.

Table 4. Machine learning selection methods and respective hyper-parameters used in this study.

Selection method	Full name of the selection method	Package and function	Hyper-parameters
CF	Conditional variable importance for random forests	party, varimp	conditional = TRUE
SRC	Random survival forests with variable importance	randomForestSRC, rfsrc	ntree = 500, nsplit = 10<br>mtry = (number of features)/3,<br>nodesize=5
SRC-VH	Random survival forests with variable hunting	randomForestSRC, var.select	method = "vh", ntree=500,<br>nodesize=5, splitrule="logrank",<br>nsplit=10, K=5
MRMR	Minimum redundancy maximum relevance filter	mRMRe, mrmr	
UM	Univariate model	mlr, various	

Supplementary Random Signature Size

According to the rule of thumb that Cox proportional hazards models should be used with a minimum of 10 events per predictor variable (EPV), we should use 2 to 16 random genes in our datasets, since TCGA and METABRIC contain the smallest and largest numbers of 17 and 164 events, respectively. However, this rule is based on two simulation studies and may be relaxed[70]. Moreover, a study investigating this rule of thumb in 2 million anonymized patient records suggested that sample size for developing prognostic models is not simply related to EPV and that EPV should be dataset dependent.

For these reasons, we randomly sampled gene sets containing a different number of genes ranging from 1-101 for each dataset. The sampling was repeated 100 times for each number of genes, Cox models were fitted, and the median C-index

was calculated. As can be seen in the top Figure 1, the prognostic power is indeed dataset dependent. For all datasets except METABRIC, we could investigate a limited number of genes, since the Cox model does not converge with a smaller number of events. Nonetheless, we see that prognostic power reaches a plateau if a sufficient number of events is considered (METABRIC). For the rest datasets, the optimal number of genes seems to be in the range of 15-25 genes. In the bottom Figure 1, this range can be inspected more closely. As can be seen, the distribution of the median C-indices increases in the range from 1-15 genes in a gene set, after which it seems to fluctuate around a constant value. Chou et. al. have shown that the optimal number of genes in a signature lies around 20 and that with a larger number of genes, a model tends to overfit data (Figure 4 in[71]). Moreover, most clinically relevant gene-expression signatures tend to contain a smaller number of genes varying from 2-50 (*Supplementary Reported Gene Selections*). Thus, we chose to sample 20 random genes in all datasets.

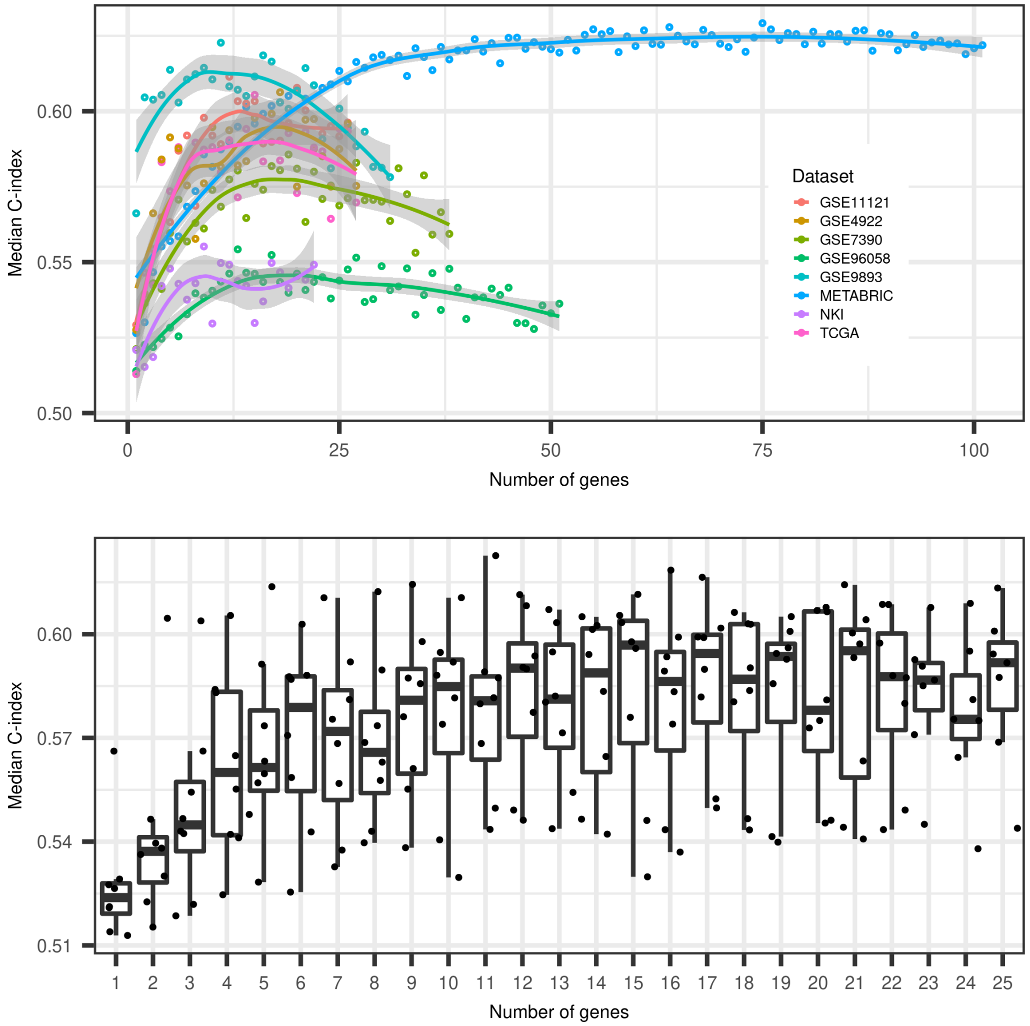

Figure 1. Relationship of median C-index and the number of genes in a random gene set. The median C-index was calculated based on 100 genes set sampled at random for each data point. The Cox proportional hazards model was used as a prognostic model. (top) Median C-indices computed based on the individual dataset. (bottom) Distribution of C-indices for all datasets in the range from 1-20 genes in a gene set.

Supplementary Random Signature Superiority

In order to examine whether Random Signature Superiority (RSS) is present in this study, we we calculated the number of random signatures performing above the C-index of the reported 26-gene signature[41] (which has roughly the same size as random signatures, see Supplementary Reported Gene Selections) for each prognostic model and averaged this value over all datasets. The results are shown in Figure 5. We found (*Supplementary Random Signature Superiority*) that more than 60% of

random signatures outperform the aforementioned reported signature in 4 of 8 datasets, exactly 49% in one dataset, and less than 22% in the remaining 3 datasets. Averaging across datasets, 44% of random signatures outperform the aforementioned reported signature.

Table 5. Evaluation of the Random Signature Superiority.

Dataset	Average [%]
GSE11121	62
GSE4922	67
GSE7390	8
GSE96058	22
GSE9893	49
METABRIC	64
NKI	19
TCGA	60
All datasets	44

Supplementary Comparison Random And Reported Signatures

In order to inspect the differences in prognostic power between random and reported selection methods, we plot the distributions in form of the violin plots for each model and each dataset in Figure 2. The distributions are compared using the Wilcoxon rank sum test. The significance levels are shown below the violin plots. As can be inspected here, reported signatures tend to have higher C-indices than random signatures, although the level of statistical significance varies across models and datasets.

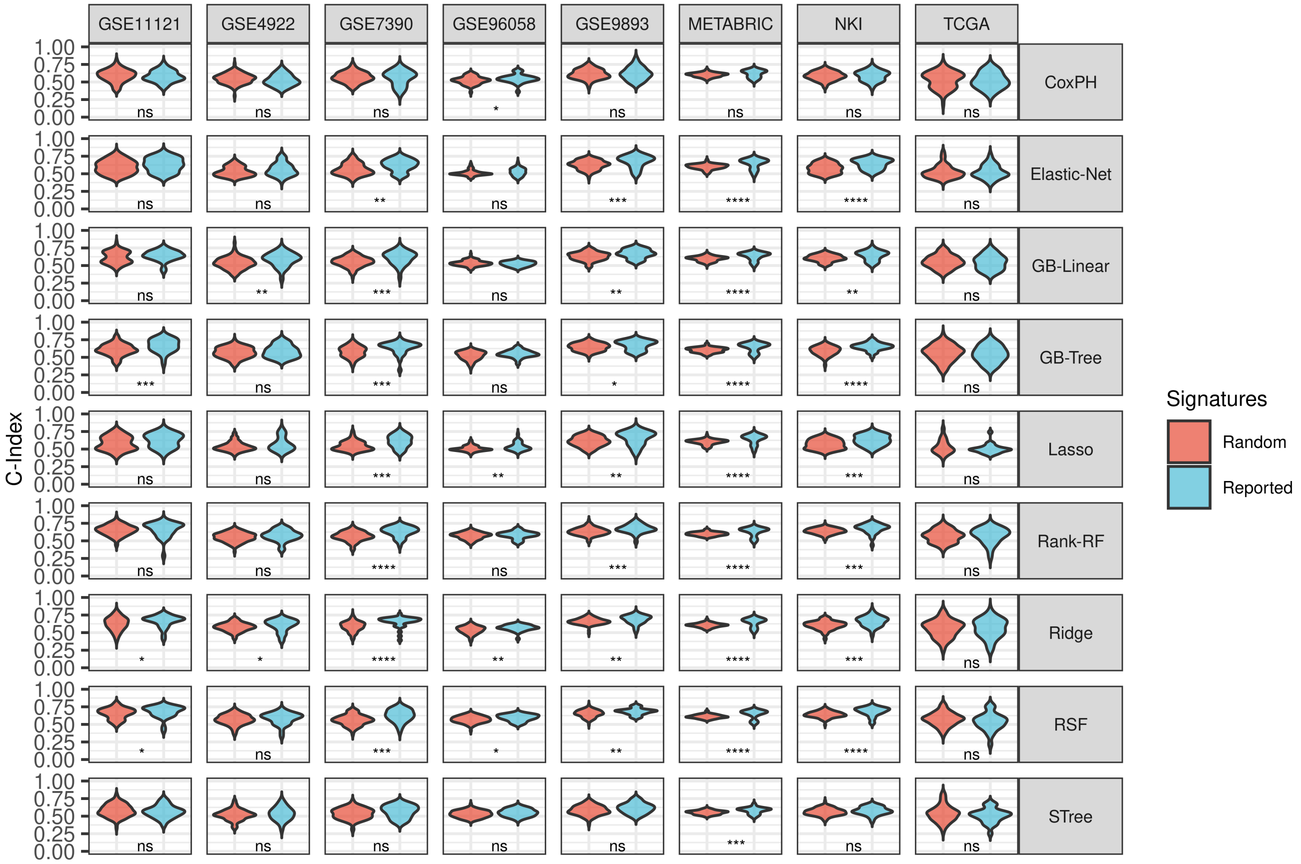

Figure 2. Comparison of C-indices for random and reported selection methods.

Supplementary Dataset Dependency

We investigated whether the median of the sample medians (MOM) and the median absolute deviation (MAD) correlates with the number of subjects as well as with the event rate in a dataset. Figures 3, 4, 5, 6 plot the results for each prognostic model along with the corresponding Spearman's rank correlation coefficients and their p-values. As can be inspected in these plots, the MOM and MAD seem to be uncorrelated with both the number of subjects and the event rate.

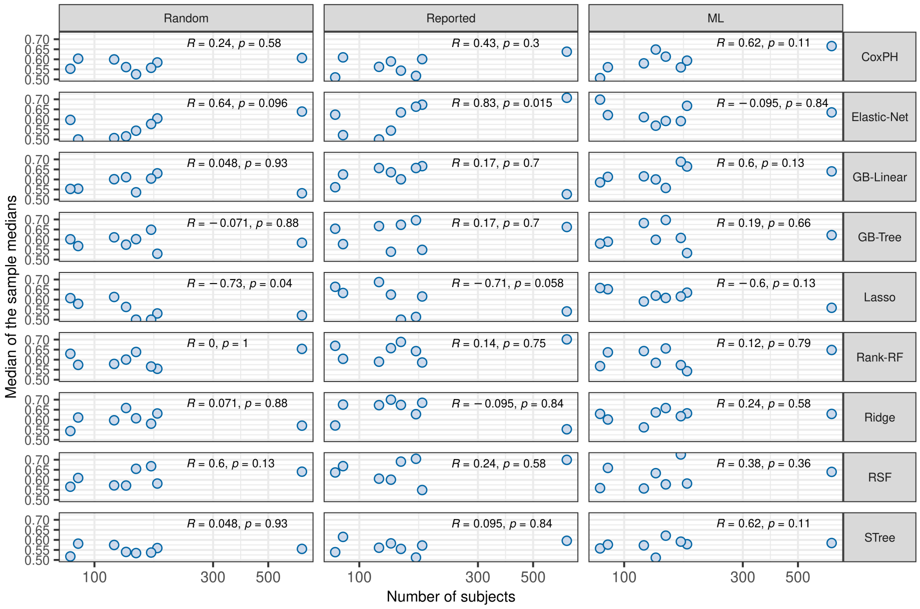

Figure 3. **Median of the sample medians in relation to the number of subjects.**

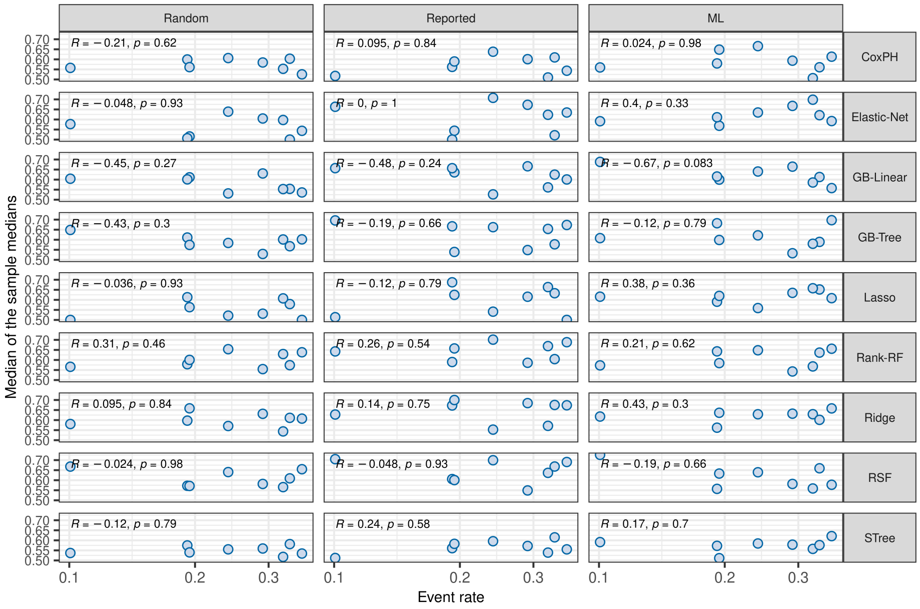

Figure 4. **Median of the sample medians in relation to the event rate.**

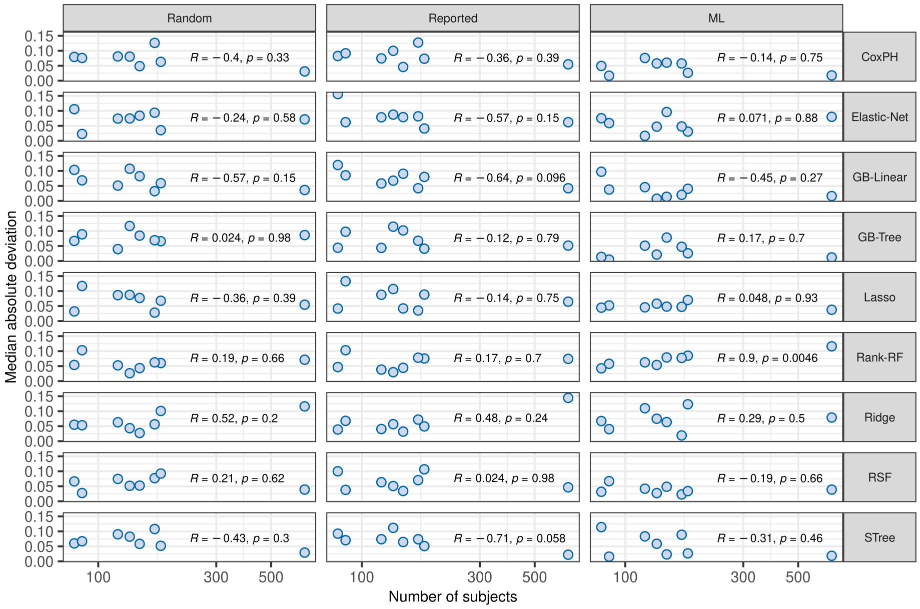

Figure 5. Median absolute deviation in relation to the number of subjects.

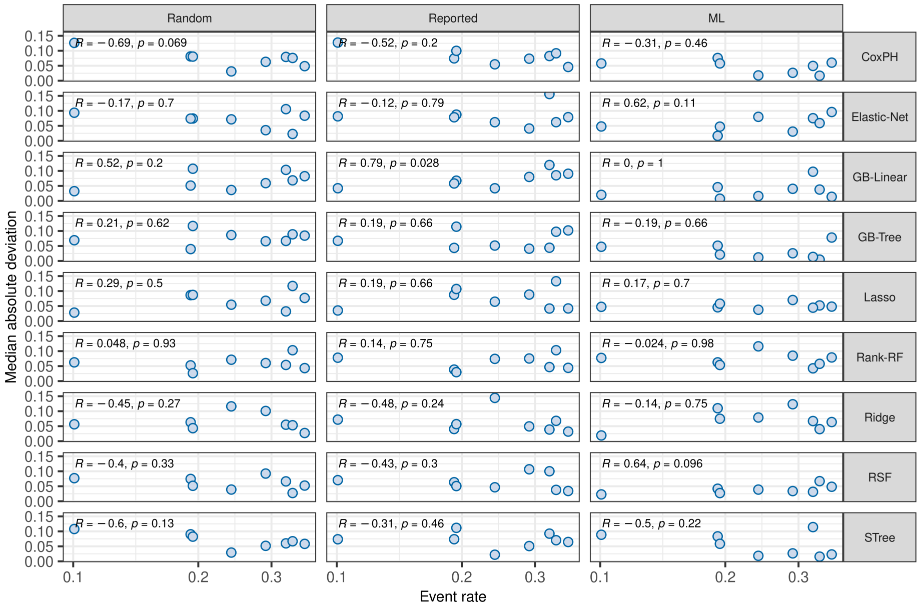

Figure 6. Median absolute deviation in relation to the event rate.

Supplementary Combined Dataset

In order to investigate large sample sizes, we combined the 8 datasets into one large dataset resulting in 2553 subjects (683+86+134+1102+120+179+80+169 = 2553). As can be seen from the number of subjects, all 1102 subjects from the GSE96058 dataset were integrated without downsampling, since the resulting event rate of 0.15 seemed to be sufficient. The datasets were standardized, normalized, and annotated as described in section *Supplementary Data*. A list of genes common to all datasets (3969 genes) was extracted in order to combine the datasets based on this list. The Z-score transformation was applied to a single dataset[72]. Subsequently, the single datasets were combined into one large dataset. The following sampling procedure was applied: 20 genes were selected at random, and the dataset identification was included as covariate in the Cox proportional hazards model in order to directly correct for batch effects and the median C-index was measured. This sampling procedure was repeated 1000 times – resulting in 1000 different random signatures – and the median of the sample medians was computed. We resampled the data with different sample sizes ranging from 800 to 2500 subjects. We kept the event rate constant (event rate = 0.15) in each sample in order to investigate the relation between sample size and prognostic performance since then we have already shown (*Supplementary Dataset Dependency*) that larger event rates do not increase prognostic power. As can be seen in Figure 7, the performance does not increase with larger sample sizes (Pearson correlation coefficient $R = 0.24, p = 0.33$).

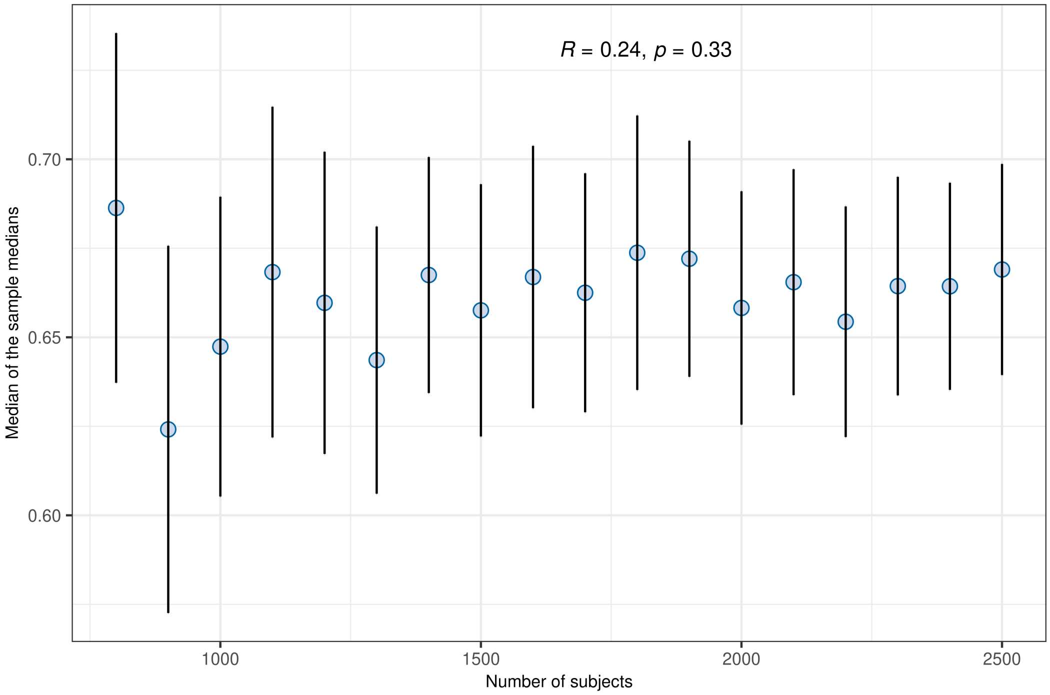

Figure 7. Median of the sample medians (MOM) of each dataset. Each data point represents the MOM computed for each prognostic model.

Supplementary Event Type

There are differences in C-indices between datasets, which may be related to the inter-platform and inter-cohort variability. In order to investigate this in more detail, we computed the median of the sample medians, i.e. the median C-index for each prognostic model, as already described above. Figure 8 shows the results for each selection method – random reported and using machine learning – for all datasets. Only GSE96058 and TCGA include the overall survival, whereas other datasets include disease-free (METABRIC), distant-metastasis-free (GSE9893, GSE7390, GSE11121), or recurrence-free survival (NKI, GSE4922). The prediction of these event types is more specific than the prediction of the overall survival, which may include events that are not related to the disease. Figure 8 shows that both GSE96058 and TCGA exhibit the lowest performances for selections from reported signatures, as well as comparably lowest performances for random selections. As expected, the differences in C-indices that follow almost the same pattern for random and reported selections to disappear in the case of selections with machine learning, since the algorithms were trained to specifically select genes that best predict the target (event): irrespective of how the target is defined.

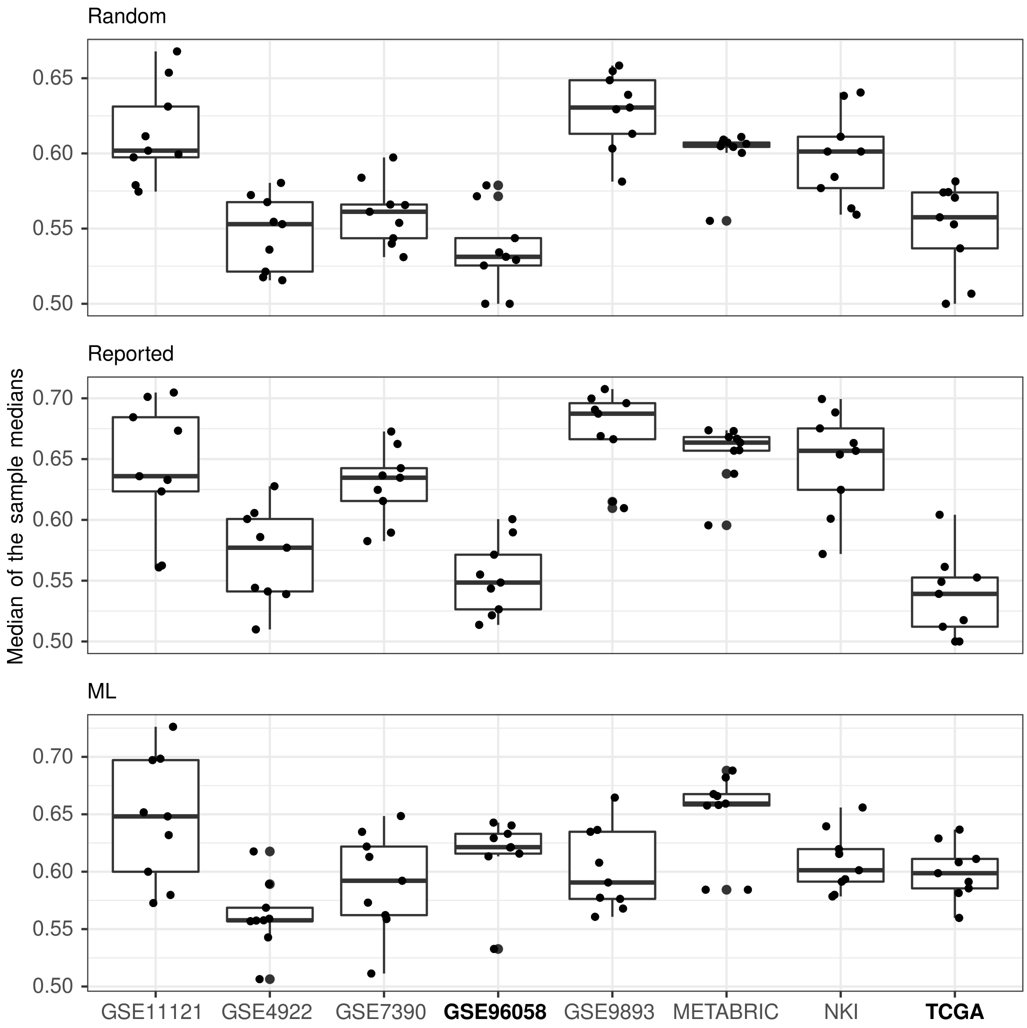

Figure 8. **Median of the sample medians (MOM) across datasets.** Each data point represents the MOM for each prognostic model. Only GSE96058 and TCGA (in bold) include the overall survival, whereas other datasets include disease-free (METABRIC), distant-metastasis-free (GSE9893, GSE7390, GSE11121), or recurrence-free survival (NKI, GSE4922). Boxplots are shown for each selection method: random selection (Random), selections from reported signatures (Reported), and selections using machine learning (ML).

Supplementary Confounding Analysis

The Effect of Age

The Table 6 compares the prognostic performance of all 9 prognostic models used in this study in predicting survival outcomes based on age as the sole variable. The C-index values in the table represent the concordance between the predicted and observed survival times. From this table, we can observe that the performance of the models varies across different datasets. For example, the Ridge model shows relatively high C-index values in datasets such as METABRIC, GSE11121, and GSE96058, indicating good predictive ability in those datasets. On the other hand, the Gradient Boosting linear model-based (GB-Linear) and Survival Tree (STree) models exhibit higher C-index values in the TCGA dataset, suggesting better performance in that specific dataset. The maximum C-index is achieved using the Stree model in the TCGA dataset. However, the overall average of the C-indices is 0.593 with the standard deviation of 0.063, indicating that prognostic models based on the age as the sole variable performs better than the prognostic models based on random signatures, while being comparable to the C-indices based on reported signatures and signatures selected with machine learning.

Table 6. Comparison of prognostic models based on the age as the sole variable. The overall average of the C-indices is 0.593 with the standard deviation of 0.063. Each row represents prognostic model used for the computation: Cox proportional hazards model (CoxPH), Lasso regression (Lasso), Ridge regression (Ridge), elastic net survival regression (Elastic-Net), Gradient boosting with linear learners (GB-Linear), with tree-based learners (GB-Tree), Random survival forests (RSF), maximally selected rank statistics random forests (Rank-RF) , and survival trees (STree).

Model	METABRIC	GSE11121	GSE96058	GSE7390	GSE9893	NKI	TCGA	GSE4922
Cox,PH_model	0.566	0.600	0.654	0.529	0.593	0.627	0.526	0.509
Ridge	0.626	0.685	0.714	0.507	0.597	0.594	0.630	0.595
Elastic-Net	0.597	0.553	0.635	0.596	0.555	0.656	0.645	0.561
Lasso	0.622	0.588	0.695	0.557	0.580	0.629	0.640	0.585
GB-Tree	0.590	0.591	0.649	0.532	0.561	0.685	0.642	0.600
GB-Linear	0.611	0.532	0.646	0.583	0.634	0.602	0.707	0.508
RSF	0.575	0.527	0.645	0.581	0.662	0.564	0.657	0.573
Rank-RF	0.587	0.534	0.564	0.553	0.667	0.566	0.731	0.571
STree	0.580	0.551	0.611	0.507	0.623	0.544	0.704	0.531

Moreover, the Table 7 presents the correlations between the scores of all 9 prognostic models and the age, computed using the Pearson correlation coefficient. The data used for the analysis is based on the METABRIC dataset. We specifically utilized the METABRIC dataset, which is the largest dataset among others, for our analysis. Each row represents a specific prognostic model, including Cox proportional hazards model, Lasso regression, Ridge regression, elastic net survival regression, gradient boosting with linear learners, gradient boosting with tree-based learners, random survival forests, maximally selected rank statistics random forests, and survival trees.

The correlation values in the table indicate the strength and direction of the relationship between the models' scores and age. A positive correlation coefficient suggests a positive association between the scores and age, meaning that as age increases, the scores tend to increase as well. Conversely, a negative correlation coefficient indicates a negative association, where higher age is associated with lower scores.

As can be clearly seen in this table, there is no significant correlation of age with models' scores. For example, the Cox proportional hazards model (CoxPH) shows a very weak negative correlation with age, with a correlation coefficient of -0.03 (for this example, see Figure 9). The overall highest correlation coefficient is 0.09 (Ridge), indicating a very weak positive relation with age. The Elastic-Net and Lasso models have no correlation with age, as indicated by correlation coefficients of 0.00. Similar interpretations can be made for the other models listed in the Table 7.

Overall, these results demonstrates that there is no associations between the prognostic models' scores and the age.

The Effect of Clinical Variables

Table 8 presents the C-index values for all 9 prognostic models based on the Nottingham Prognostic Index (NPI) in the METABRIC dataset. Each raw in the table represents a specific prognostic model, such as Cox proportional hazards model (CoxPH), Lasso regression (Lasso), Ridge regression (Ridge), elastic net survival regression (Elastic-Net), Gradient boosting with linear learners (GB-Linear), Gradient boosting with tree-based learners (GB-Tree), Random survival forests (RSF), maximally selected rank statistics random forests (Rank-RF), and survival trees (STree).

Table 7. Correlations of models' scores with age. The correlations were computed using the Pearson correlation. The results are based on the METABRIC dataset. We specifically utilized the METABRIC dataset, which is the largest dataset among others. Each row represents prognostic model used for the computation: Cox proportional hazards model (CoxPH), Lasso regression (Lasso), Ridge regression (Ridge), elastic net survival regression (Elastic-Net), Gradient boosting with linear learners (GB-Linear), with tree-based learners (GB-Tree), Random survival forests (RSF), maximally selected rank statistics random forests (Rank-RF) , and survival trees (STree). Each column represents selection method used for the computation: random selection (Random), using reported signatures (Reported), and selecting with machine learning (ML).

Model	Random	Reported	ML
CoxPH	-0.03	-0.05	0.01
Ridge	0.09	-0.09	0.01
Elastic-Net	0.00	0.00	0.00
Lasso	0.00	0.00	0.00
GB-Tree	0.01	-0.04	0.05
GB-Linear	0.04	0.02	-0.04
RSF	0.00	-0.03	-0.03
Rank-RF	0.01	-0.05	0.05
Survival_Tree	0.00	0.00	-0.08

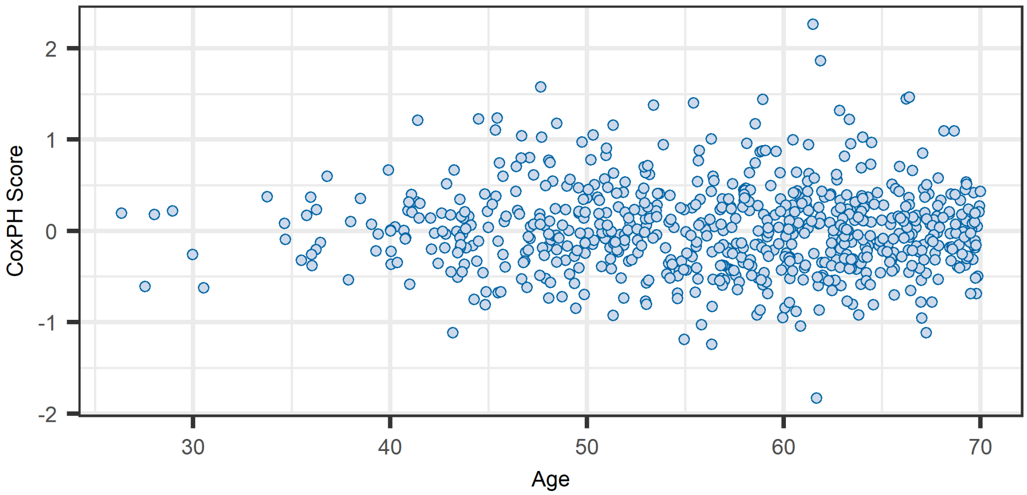

Figure 9. Exemplary plot of age vs. Cox proportional hazards model scores showing a correlation coefficient of -0.03. The scatter plot demonstrates the relation between age and the scores generated by the Cox proportional hazards model, indicating a weak negative correlation between the two variables.

Comparing the first column (METABRIC) of Table 7 with the performance values in Table 8, it becomes evident that each of the prognostic models tends to perform better when utilizing the NPI compared to when using age as the sole variable.

The CoxPH model's performance increased from a C-index of 0.566 to 0.69. Similarly, the Ridge regression model demonstrated a significant improvement, going from a C-index of 0.626 to 0.69. Performance enhancements were also visible in the Elastic-Net survival regression (from 0.597 to 0.65), Lasso regression (from 0.622 to 0.68), GB-Tree (from 0.590 to 0.69), and GB-Linear (from 0.611 to 0.67) models. Interestingly, Random Survival Forests (RSF) and Rank-RF showed a modest improvement when utilizing the NPI compared to age alone, increasing from 0.575 to 0.65 and 0.587 to 0.63, respectively. The performance of Survival Trees (STree), however, saw only a minimal increase from a C-index of 0.580 (Table 1) to 0.61 (Table 2).

In conclusion, both – age and the Nottingham Prognostic Index – have a significant effect on the performance of various prognostic models and can be used as a baseline for models based on gene expression signatures. Crucially, the clinical variables encapsulated within the Nottingham Prognostic Index demonstrated considerable performance with C-indices around 0.67 (Table 8). This substantiates their robust applicability for prognostic purposes in this dataset.

Table 8. The performance of prognostic models based on the Nottingham Prognostic Index. C-indices were computed based on the METABRIC dataset, which is the largest dataset among others. Cox proportional hazards model (CoxPH), Lasso regression (Lasso), Ridge regression (Ridge), elastic net survival regression (Elastic-Net), Gradient boosting with linear learners (GB-Linear), with tree-based learners (GB-Tree), Random survival forests (RSF), maximally selected rank statistics random forests (Rank-RF), and survival trees (STree).

	C-index
CoxPH	0.69
Ridge	0.69
Elastic-Net	0.65
Lasso	0.68
GB-Tree	0.69
GB-Linear	0.67
RSF	0.65
Rank-RF	0.63
STree	0.61
Median	0.67

Comparison With Other Measures

For completeness, we computed the area under the curve (AUC), the Pearson correlation and the Spearman correlation coefficients using randomly selected signatures in the METABRIC dataset (see Figure 10). Figure 10 demonstrates that one could alternatively use the AUC instead of the C-index, and the results remain consistent when using the AUC as the performance measure. In order to compare the measures, we specifically utilized random signatures in our analysis. The AUC and C-index are both statistical measures used to evaluate the performance of prognostic models. The AUC is a scalar value that ranges from 0 to 1, where 1 represents a perfect model, 0.5 is equivalent to random guessing, and 0 represents a perfectly incorrect model. Harrell's C-index, on the other hand, is a more general measure that can be used for both binary and survival outcomes. It estimates the probability that for a randomly chosen pair of subjects, the one who experienced the event first had a higher predicted probability of experiencing the event.

The AUC and C-index are both measures of rank correlation, which measures how well a model can order individuals by risk. For binary outcomes, the C-index is equivalent to the AUC. However, for survival data, where censored observations are present, the C-index is often a more appropriate measure. This is because it takes censoring into account, providing a more accurate reflection of the model's predictive performance in these contexts. Nevertheless, in Figure 10 we see a strong association of between the C-index and AUC values.

Regarding the Pearson and Spearman correlation coefficients, it is obvious from Figure 10 that they do not approach high values close to one and are weakly associated with the C-index values. When it comes to evaluating the performance of a survival model, these measures do not handle censored observations. Thus, they may not provide an accurate reflection of the performance of survival models. Also, the predicted risk score is a measure of the risk of event occurrence, rather than a prediction of the actual survival time. Consequently, correlating the actual survival times with predicted risk scores might not yield meaningful results because they are not measuring the same thing.

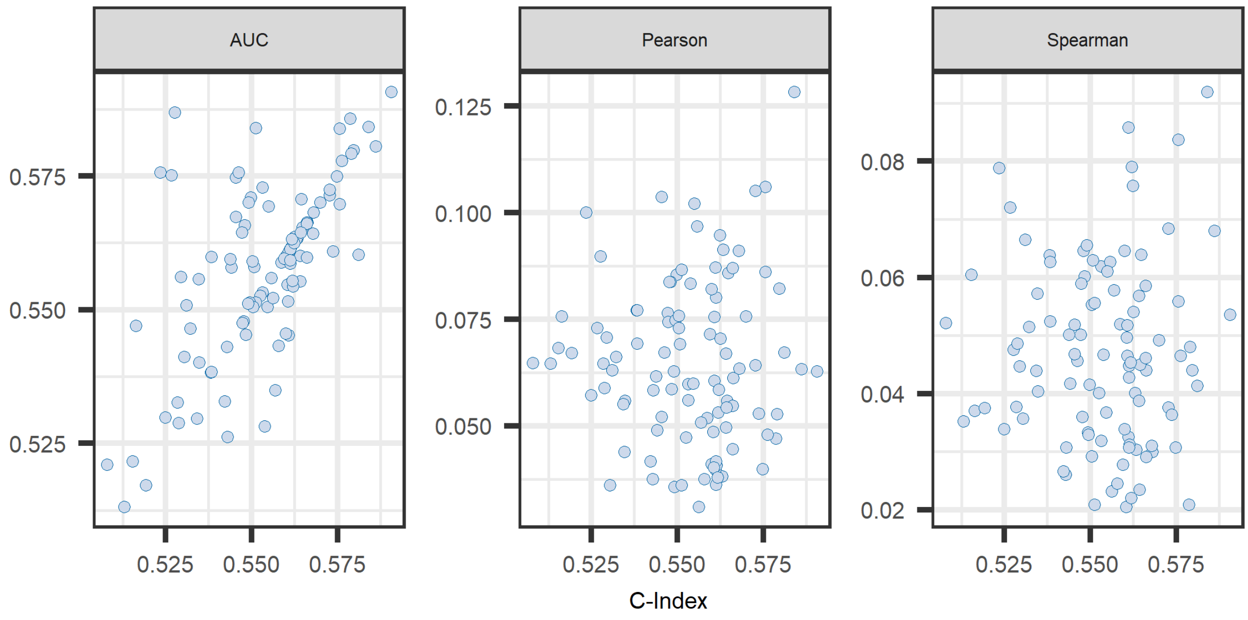

Figure 10. Comparison of the C-index with the Area Under the Curve, Pearson and Spearman correlation coefficients. In order to compare the measures, we specifically utilized random signatures in the METABRIC dataset.

References

1. Barrett, T. *et al.* Ncbi geo: mining millions of expression profiles—database and tools. *Nucleic acids research* **33**, D562–D566 (2005).
2. Davis, S. & Meltzer, P. S. Geoquery: a bridge between the gene expression omnibus (geo) and bioconductor. *Bioinformatics* **23**, 1846–1847 (2007).
3. Irizarry, R. A. *et al.* Exploration, normalization, and summaries of high density oligonucleotide array probe level data. *Biostatistics* **4**, 249–264 (2003).
4. Langfelder, P. & Horvath, S. Wgcna: an r package for weighted correlation network analysis. *BMC bioinformatics* **9**, 1–13 (2008).
5. Leek, J. T., Johnson, W. E., Parker, H. S., Jaffe, A. E. & Storey, J. D. The sva package for removing batch effects and other unwanted variation in high-throughput experiments. *Bioinformatics* **28**, 882–883 (2012).
6. Curtis, C. *et al.* The genomic and transcriptomic architecture of 2,000 breast tumours reveals novel subgroups. *Nature* **486**, 346–352, DOI: 10.1038/nature10983 (2012).
7. Ritchie, M. E. *et al.* Limma powers differential expression analyses for RNA-sequencing and microarray studies. *Nucleic Acids Res.* **43**, e47, DOI: 10.1093/nar/gkv007 (2015).
8. Dunning, M., Lynch, A. & Eldridge, M. illuminahumanv4. db: Illumina humanht12v4 annotation data (chip illuminahumanv4). *R package version* **1** (2015).
9. Colaprico, A. *et al.* Tcgabiolinks: an r/bioconductor package for integrative analysis of tcga data. *Nucleic acids research* **44**, e71–e71 (2016).
10. Robinson, M. D., McCarthy, D. J. & Smyth, G. K. edger: a bioconductor package for differential expression analysis of digital gene expression data. *Bioinformatics* **26**, 139–140 (2010).
11. Law, C. W., Chen, Y., Shi, W. & Smyth, G. K. voom: Precision weights unlock linear model analysis tools for rna-seq read counts. *Genome biology* **15**, 1–17 (2014).
12. Schmidt, M. *et al.* The humoral immune system has a key prognostic impact in node-negative breast cancer. *Cancer Res.* **68**, 5405–5413, DOI: 10.1158/0008-5472.CAN-07-5206 (2008).

13. Brueffer, C. *et al.* Clinical Value of RNA Sequencing–Based Classifiers for Prediction of the Five Conventional Breast Cancer Biomarkers: A Report From the Population-Based Multicenter Sweden Cancerome Analysis Network—Breast Initiative. *JCO Precis. Oncol.* 1–18, DOI: 10.1200/po.17.00135 (2018).

14. Ivshina, A. V. *et al.* Genetic reclassification of histologic grade delineates new clinical subtypes of breast cancer. *Cancer Res.* **66**, 10292–10301, DOI: 10.1158/0008-5472.CAN-05-4414 (2006).

15. Desmedt, C. *et al.* Strong time dependence of the 76-gene prognostic signature for node-negative breast cancer patients in the TRANSBIG multicenter independent validation series. *Clin. Cancer Res.* **13**, 3207–3214, DOI: 10.1158/1078-0432.CCR-06-2765 (2007).

16. Chanrion, M. *et al.* A gene expression signature that can predict the recurrence of tamoxifen-treated primary breast cancer. *Clin. Cancer Res.* **14**, 1744–1752, DOI: 10.1158/1078-0432.CCR-07-1833 (2008).

17. Van De Vijver, M. J. *et al.* A gene-expression signature as a predictor of survival in breast cancer. *New Engl. J. Medicine* **347**, 1999–2009 (2002).

18. van't Veer, L. J. *et al.* Expression profiling predicts outcome in breast cancer. *Breast Cancer Res.* **5**, 1–2 (2002).

19. Huang, S., Murphy, L. & Xu, W. Genes and functions from breast cancer signatures. *BMC cancer* **18**, 1–15 (2018).

20. Carlson, M., Falcon, S., Pages, H. & Li, N. org. hs. eg. db: Genome wide annotation for human. *R package version* **3**, 3 (2019).

21. Obayashi, T. *et al.* Coxpresdb: a database of coexpressed gene networks in mammals. *Nucleic acids research* **36**, D77–D82 (2007).

22. Rody, A. *et al.* A clinically relevant gene signature in triple negative and basal-like breast cancer. *Breast cancer research* **13**, 1–12 (2011).

23. Ma, X.-J. *et al.* A five-gene molecular grade index and hoxb13: Il17br are complementary prognostic factors in early stage breast cancer. *Clin. cancer research* **14**, 2601–2608 (2008).

24. Liu, J. *et al.* Identification of a gene signature in cell cycle pathway for breast cancer prognosis using gene expression profiling data. *BMC medical genomics* **1**, 1–12 (2008).

25. Chang, J. C. *et al.* Gene expression profiling for the prediction of therapeutic response to docetaxel in patients with breast cancer. *The Lancet* **362**, 362–369 (2003).

26. Filipits, M. *et al.* A new molecular predictor of distant recurrence in er-positive, her2-negative breast cancer adds independent information to conventional clinical risk factors. *Clin. Cancer Res.* **17**, 6012–6020 (2011).

27. Minuti, G. *et al.* Increased met and hgf gene copy numbers are associated with trastuzumab failure in her2-positive metastatic breast cancer. *Br. journal cancer* **107**, 793–799 (2012).

28. Toussaint, J. *et al.* Improvement of the clinical applicability of the genomic grade index through a qrt-pcr test performed on frozen and formalin-fixed paraffin-embedded tissues. *BMC genomics* **10**, 1–13 (2009).

29. Sotiriou, C. *et al.* Gene expression profiling in breast cancer: understanding the molecular basis of histologic grade to improve prognosis. *J. Natl. Cancer Inst.* **98**, 262–272 (2006).

30. Staaf, J. *et al.* Identification of subtypes in human epidermal growth factor receptor 2–positive breast cancer reveals a gene signature prognostic of outcome. *J. Clin. Oncol.* **28**, 1813–1820 (2010).

31. Ma, X.-J. *et al.* The hoxb13: Il17br expression index is a prognostic factor in early-stage breast cancer. *J. clinical oncology* **24**, 4611–4619 (2006).

32. Liu, R. *et al.* The prognostic role of a gene signature from tumorigenic breast-cancer cells. *New Engl. J. Medicine* **356**, 217–226 (2007).

33. Cuzick, J. *et al.* Prognostic value of a combined estrogen receptor, progesterone receptor, ki-67, and human epidermal growth factor receptor 2 immunohistochemical score and comparison with the genomic health recurrence score in early breast cancer. *J Clin Oncol* **29**, 4273–4278 (2011).

34. Teschendorff, A. E., Miremadi, A., Pinder, S. E., Ellis, I. O. & Caldas, C. An immune response gene expression module identifies a good prognosis subtype in estrogen receptor negative breast cancer. *Genome biology* **8**, 1–16 (2007).

35. Iwao-Koizumi, K. *et al.* Prediction of docetaxel response in human breast cancer by gene expression profiling. *J. clinical oncology* **23**, 422–431 (2005).

36. Minn, A. J. *et al.* Genes that mediate breast cancer metastasis to lung. *Nature* **436**, 518–524 (2005).

37. Karn, T. *et al.* Melanoma antigen family a identified by the bimodality index defines a subset of triple negative breast cancers as candidates for immune response augmentation. *Eur. J. Cancer* **48**, 12–23 (2012).

38. Sabatier, R. *et al.* A gene expression signature identifies two prognostic subgroups of basal breast cancer. *Breast cancer research treatment* **126**, 407–420 (2011).

39. Tutt, A. *et al.* Risk estimation of distant metastasis in node-negative, estrogen receptor-positive breast cancer patients using an rt-pcr based prognostic expression signature. *BMC cancer* **8**, 1–15 (2008).

40. Yau, C. *et al.* A multigene predictor of metastatic outcome in early stage hormone receptor-negative and triple-negative breast cancer. *Breast cancer research* **12**, 1–15 (2010).

41. Karn, T. *et al.* Homogeneous datasets of triple negative breast cancers enable the identification of novel prognostic and predictive signatures. *PloS one* **6**, e28403 (2011).

42. Thuerigen, O. *et al.* Gene expression signature predicting pathologic complete response with gemcitabine, epirubicin, and docetaxel in primary breast cancer. *J Clin Oncol* **24**, 1839–1845 (2006).

43. Miller, L. D. *et al.* An expression signature for p53 status in human breast cancer predicts mutation status, transcriptional effects, and patient survival. *Proc. Natl. Acad. Sci.* **102**, 13550–13555 (2005).

44. Sørlie, T. *et al.* Repeated observation of breast tumor subtypes in independent gene expression data sets. *Proc. national academy sciences* **100**, 8418–8423 (2003).

45. Pawitan, Y. *et al.* Gene expression profiling spares early breast cancer patients from adjuvant therapy: derived and validated in two population-based cohorts. *Breast cancer research* **7**, 1–12 (2005).

46. van Vliet, M. H. *et al.* Pooling breast cancer datasets has a synergetic effect on classification performance and improves signature stability. *BMC genomics* **9**, 1–22 (2008).

47. Paik, S. *et al.* A multigene assay to predict recurrence of tamoxifen-treated, node-negative breast cancer. *New Engl. J. Medicine* **351**, 2817–2826 (2004).

48. Finak, G. *et al.* Stromal gene expression predicts clinical outcome in breast cancer. *Nat. medicine* **14**, 518–527 (2008).

49. Sánchez-Navarro, I. *et al.* An 8-gene qrt-pcr-based gene expression score that has prognostic value in early breast cancer. *BMC cancer* **10**, 1–10 (2010).

50. Rody, A. *et al.* T-cell metagene predicts a favorable prognosis in estrogen receptor-negative and her2-positive breast cancers. *Breast Cancer Res.* **11**, 1–13 (2009).

51. Végran, F. *et al.* Gene expression profile and response to trastuzumab–docetaxel-based treatment in breast carcinoma. *Br. journal cancer* **101**, 1357–1364 (2009).

52. Wang, Y. *et al.* Gene-expression profiles to predict distant metastasis of lymph-node-negative primary breast cancer. *The Lancet* **365**, 671–679 (2005).

53. Bischl, B. *et al.* mlr: Machine learning in r. *The J. Mach. Learn. Res.* **17**, 5938–5942 (2016).

54. Cox, D. R. Regression models and life-tables. *J. Royal Stat. Soc. Ser. B (Methodological)* **34**, 187–202 (1972).

55. Zou, H. & Hastie, T. Regularization and variable selection via the elastic net. *J. royal statistical society: series B (statistical methodology)* **67**, 301–320 (2005).

56. Tibshirani, R. The lasso method for variable selection in the cox model. *Stat. medicine* **16**, 385–395 (1997).

57. Simon, N., Friedman, J., Hastie, T. & Tibshirani, R. Regularization paths for cox's proportional hazards model via coordinate descent. *J. statistical software* **39**, 1 (2011).

58. Schapire, R. E. The strength of weak learnability. *Mach. learning* **5**, 197–227 (1990).

59. Friedman, J., Hastie, T. & Tibshirani, R. Additive logistic regression: a statistical view of boosting (with discussion and a rejoinder by the authors). *The annals statistics* **28**, 337–407 (2000).

60. Hothorn, T., Bühlmann, P., Dudoit, S., Molinaro, A. & Van Der Laan, M. J. Survival ensembles. *Biostatistics* **7**, 355–373 (2006).

61. Friedman, J. H. Stochastic gradient boosting. *Comput. statistics & data analysis* **38**, 367–378 (2002).

62. Gordon, L. & Olshen, R. A. Tree-structured survival analysis. *Cancer treatment reports* **69**, 1065–1069 (1985).

63. Ishwaran, H., Kogalur, U. B., Blackstone, E. H. & Lauer, M. S. Random survival forests. *The annals applied statistics* **2**, 841–860 (2008).

64. Morgan, J. N. & Sonquist, J. A. Problems in the analysis of survey data, and a proposal. *J. Am. statistical association* **58**, 415–434 (1963).

65. Breiman, L., Friedman, J. H., Olshen, R. A. & Stone, C. J. *Classification and regression trees* (Routledge, 2017).

66. Breiman, L. Random forests. *Mach. learning* **45**, 5–32 (2001).

67. Hothorn, T., Hornik, K. & Zeileis, A. Unbiased recursive partitioning: A conditional inference framework. *J. Comput. Graph. statistics* **15**, 651–674 (2006).

68. Lausen, B. & Schumacher, M. Maximally selected rank statistics. *Biometrics* 73–85 (1992).

69. Peng, H., Long, F. & Ding, C. Feature selection based on mutual information criteria of max-dependency, max-relevance, and min-redundancy. *IEEE Transactions on pattern analysis machine intelligence* **27**, 1226–1238 (2005).

70. Vittinghoff, E. & McCulloch, C. E. Relaxing the Rule of Ten Events per Variable in Logistic and Cox Regression. *Am. J. Epidemiol.* **165**, 710–718, DOI: 10.1093/aje/kwk052 (2006). https://academic.oup.com/aje/article-pdf/165/6/710/140367/kwk052.pdf.

71. Chou, H.-L. *et al.* Gene expression profiling of breast cancer survivability by pooled cdna microarray analysis using logistic regression, artificial neural networks and decision trees. *BMC bioinformatics* **14**, 1–11 (2013).

72. Cheadle, C., Vawter, M. P., Freed, W. J. & Becker, K. G. Analysis of microarray data using z score transformation. *The J. molecular diagnostics* **5**, 73–81 (2003).
